# Supplementary material for: Assessing the Causal Association between Biological Aging Biomarkers and the Development of Cerebral Small Vessel Disease: A Mendelian Randomization Study
Source: Biology (Basel). 2023 Apr 27;12(5):660. doi: 10.3390/biology12050660 (PMC10215078; doi:10.3390/biology12050660)
Supplement: Supplementary file 1 [file biology-12-00660-s001.zip › biology-2252843-supplementary.pdf]

**SUPPLEMENTAL MATERIAL**

**Catalog**

Supplement 1. GWAS datasets used in the present MR analysis................................3
Supplement 2. The single SNP analysis and leave-one-out analysis for exposures on outcomes.........................................................................................................4
Supplement 2 Figure 1. The single SNP analysis and leave-one-out analysis for LTL on WMH volume ...........................................................................4
Supplement 2 Figure 2. The single SNP analysis and leave-one-out analysis for LTL on FA ...........................................................................................5
Supplement 2 Figure 3. The single SNP analysis and leave-one-out analysis for LTL on MD...........................................................................................6
Supplement 2 Figure 4. The single SNP analysis and leave-one-out analysis for LTL on lacunar stroke ...........................................................................7
Supplement 2 Figure 5. The single SNP analysis and leave-one-out analysis for LTL on all location BMB ........................................................................9
Supplement 2 Figure 6. The single SNP analysis and leave-one-out analysis for LTL on lobar BMB ..............................................................................9
Supplement 2 Figure 7. The single SNP analysis and leave-one-out analysis for LTL on mixed or deep BMB ......................................................................10
Supplement 2 Figure 8. The single SNP analysis and leave-one-out analysis for LTL on all location ICH or SVS ...........................................................11
Supplement 2 Figure 9. The single SNP analysis and leave-one-out analysis for LTL on lobar ICH or SVS.........................................................................12
Supplement 2 Figure 10. The single SNP analysis and leave-one-out analysis for LTL on non-lobar ICH or SVS...............................................................13
Supplement 2 Figure 11. The single SNP analysis and leave-one-out analysis for GrimAge on WMH volume..............................................................................14
Supplement 2 Figure 12. The single SNP analysis and leave-one-out analysis for GrimAge on FA.........................................................................................15
Supplement 2 Figure 13. The single SNP analysis and leave-one-out analysis for GrimAge on MD .......................................................................................16
Supplement 2 Figure 14. The single SNP analysis and leave-one-out analysis for GrimAge on lacunar stroke ..........................................................................17
Supplement 2 Figure 15. The single SNP analysis and leave-one-out analysis for GrimAge on all location BMB ......................................................................18
Supplement 2 Figure 16. The single SNP analysis and leave-one-out analysis for GrimAge on lobar BMB.........................................................................19
Supplement 2 Figure 17. The single SNP analysis and leave-one-out analysis for GrimAge on mixed or deep BMB .................................................................20
Supplement 2 Figure 18. The single SNP analysis and leave-one-out analysis for GrimAge on all location ICH or SVS..............................................................21
Supplement 2 Figure 19. The single SNP analysis and leave-one-out analysis for GrimAge on lobar ICH or SVS .....................................................................22
Supplement 2 Figure 20. The single SNP analysis and leave-one-out analysis for

GrimAge on non-lobar ICH or SVS ....................................................................23
Supplement 2 Figure 21. The single SNP analysis and leave-one-out analysis for PhenoAge on WMH volume ....................................................................24
Supplement 2 Figure 22. The single SNP analysis and leave-one-out analysis for PhenoAge on FA ....................................................................25
Supplement 2 Figure 23. The single SNP analysis and leave-one-out analysis for PhenoAge on MD ....................................................................26
Supplement 2 Figure 24. The single SNP analysis and leave-one-out analysis for PhenoAge on lacunar stroke ....................................................................27
Supplement 2 Figure 25. The single SNP analysis and leave-one-out analysis for PhenoAge on all location BMB ....................................................................28
Supplement 2 Figure 26. The single SNP analysis and leave-one-out analysis for PhenoAge on lobar BMB ....................................................................29
Supplement 2 Figure 27. The single SNP analysis and leave-one-out analysis for PhenoAge on mixed or deep BMB ....................................................................30
Supplement 2 Figure 28. The single SNP analysis and leave-one-out analysis for PhenoAge on all location ICH or SVS ....................................................................31
Supplement 2 Figure 29. The single SNP analysis and leave-one-out analysis for PhenoAge on lobar ICH or SVS ....................................................................32
Supplement 2 Figure 30. The single SNP analysis and leave-one-out analysis for PhenoAge on non-lobar ICH or SVS ....................................................................33
Supplement 2 Figure 31. The single SNP analysis and leave-one-out analysis for HannumAge on WMH volume ....................................................................34
Supplement 2 Figure 32. The single SNP analysis and leave-one-out analysis for HannumAge on FA ....................................................................35
Supplement 2 Figure 33. The single SNP analysis and leave-one-out analysis for HannumAge on MD ....................................................................36
Supplement 2 Figure 34. The single SNP analysis and leave-one-out analysis for HannumAge on lacunar stroke ....................................................................37
Supplement 2 Figure 35. The single SNP analysis and leave-one-out analysis for HannumAge on all location BMB ....................................................................38
Supplement 2 Figure 36. The single SNP analysis and leave-one-out analysis for HannumAge on lobar BMB ....................................................................39
Supplement 2 Figure 37. The single SNP analysis and leave-one-out analysis for HannumAge on mixed or deep BMB ....................................................................40
Supplement 2 Figure 38. The single SNP analysis and leave-one-out analysis for HannumAge on all location ICH or SVS ....................................................................41
Supplement 2 Figure 39. The single SNP analysis and leave-one-out analysis for HannumAge on lobar ICH or SVS ....................................................................42
Supplement 2 Figure 40. The single SNP analysis and leave-one-out analysis for HannumAge on non-lobar ICH or SVS ....................................................................43
Supplement 2 Figure 41. The single SNP analysis and leave-one-out analysis for Intrinsic HorvathAge acceleration on WMH volume ....................................................................44
Supplement 2 Figure 42. The single SNP analysis and leave-one-out analysis for

Intrinsic HorvathAge acceleration on FA........................................................45
Supplement 2 Figure 43. The single SNP analysis and leave-one-out analysis for Intrinsic HorvathAge acceleration on MD........................................................47
Supplement 2 Figure 44. The single SNP analysis and leave-one-out analysis for Intrinsic HorvathAge acceleration on lacunar stroke........................................48
Supplement 2 Figure 45. The single SNP analysis and leave-one-out analysis for Intrinsic HorvathAge acceleration on all location BMB....................................48
Supplement 2 Figure 46. The single SNP analysis and leave-one-out analysis for Intrinsic HorvathAge acceleration on lobar BMB..............................................49
Supplement 2 Figure 47. The single SNP analysis and leave-one-out analysis for Intrinsic HorvathAge acceleration on mixed or deep BMB................................50
Supplement 2 Figure 48. The single SNP analysis and leave-one-out analysis for Intrinsic HorvathAge acceleration on all location ICH or SVS..........................51
Supplement 2 Figure 49. The single SNP analysis and leave-one-out analysis for Intrinsic HorvathAge acceleration on lobar ICH or SVS...................................52
Supplement 2 Figure 50. The single SNP analysis and leave-one-out analysis for Intrinsic HorvathAge acceleration on non-lobar ICH or SVS.............................53
Supplement 3. Results of other four MR methods for causal effect of exposures on outcomes........................................................................................54
Supplement 3 Table 1. Results of other four MR methods for causal effect of LTL on CSVD........................................................................................54
Supplement 3 Table 2. Results of other four MR methods for causal effect of GrimAge on CSVD........................................................................................56
Supplement 3 Table 3. Results of other four MR methods for causal effect of PhenoAge on CSVD........................................................................................57
Supplement 3 Table 4. Results of other four MR methods for causal effect of HannumAge on CSVD........................................................................................58
Supplement 3 Table 5. Results of other four MR methods for causal effect of Intrinsic HorvathAge acceleration on CSVD......................................................59
Supplement 4. Results of sensitivity analysis..........................................................60
Supplement 4 Table 1. Results of sensitivity analysis of LTL on CSVD ...........60
Supplement 4 Table 2. Results of sensitivity analysis of GrimAge on CSVD.....61
Supplement 4 Table 3. Results of sensitivity analysis of PhenoAge on CSVD ...61
Supplement 4 Table 4. Results of sensitivity analysis of HannumAge on CSVD62
Supplement 4 Table 5. Results of sensitivity analysis of Intrinsic HorvathAge acceleration on CSVD........................................................................62

**Supplement 1.** GWAS data sets used in the present MR analysis

| Traits | PubMed ID | Population （Proportion） | Sample size or Cases/Controls |
|---|---|---|---|
| LTL | 34611362 | European (94%) | 472174 |
| GrimAge | 34187551 | European | 34710 |
| PhenoAge | 34187551 | European | 34710 |
| HannumAge | 34187551 | European | 34710 |
| Intrinsic HorvathAge acceleration | 34187551 | European | 34710 |
| WMH volume | 32358547 | European | 18381 |
| FA | 32358547 | European | 17663 |
| MD | 32358547 | European | 17467 |
| lacunar stroke | 33773637 | European | 6030/248929 |
| all location BMB | 32913026 | European (94%) | 3556/25806 |
| lobar BMB | 32913026 | European (94%) | 2179/25806 |
| mixed or deep BMB | 32913026 | European (94%) | 1293/25806 |
| all location ICH or SVS | 31430377 | European | 6255/233058 |
| lobar ICH or SVS | 31430377 | European | 5240/233058 |
| non-lobar ICH or SVS | 31430377 | European | 5500/233058 |

**Supplement 2.** The single SNP analysis and leave-one-out analysis for exposures on outcomes

Supplement 2 Figure 1. The single SNP analysis and leave-one-out analysis for LTL on WMH volume

(a) Forest plot of single SNP MR

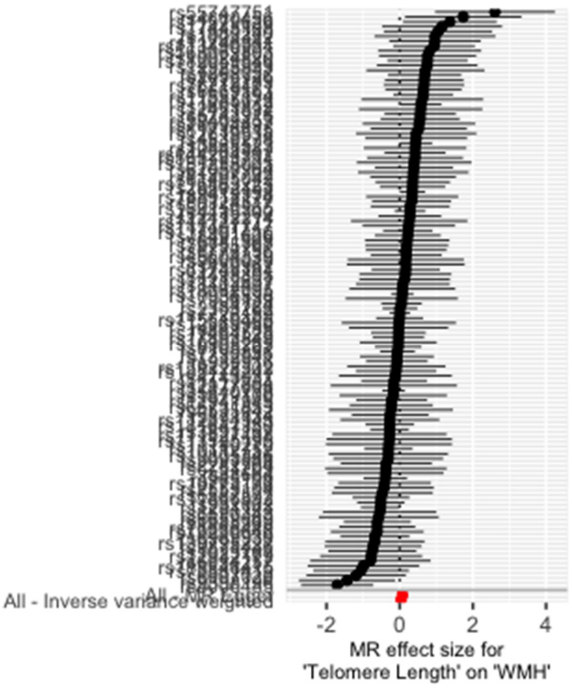


(b) Leave-one-out analysis

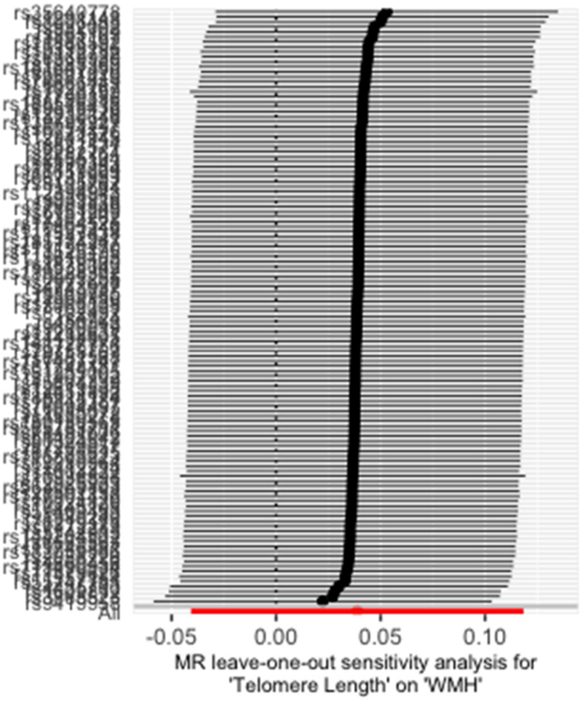


Supplement 2 Figure 2. The single SNP analysis and leave-one-out analysis for LTL on FA

(a) Forest plot of single SNP MR

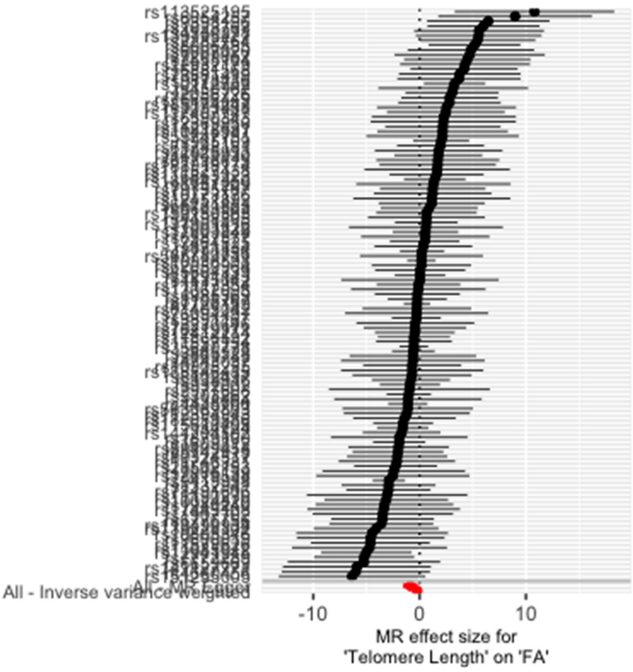


(b) Leave-one-out analysis

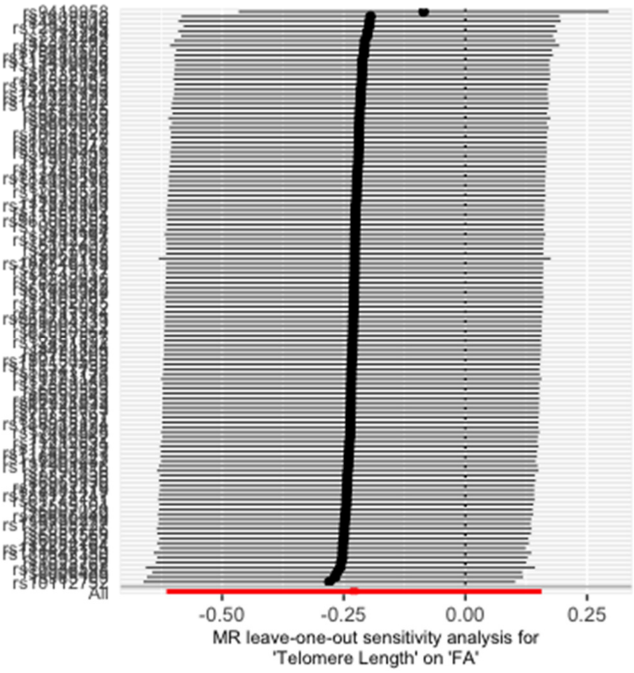


Supplement 2 Figure 3. The single SNP analysis and leave-one-out analysis for LTL on MD

(a) Forest plot of single SNP MR

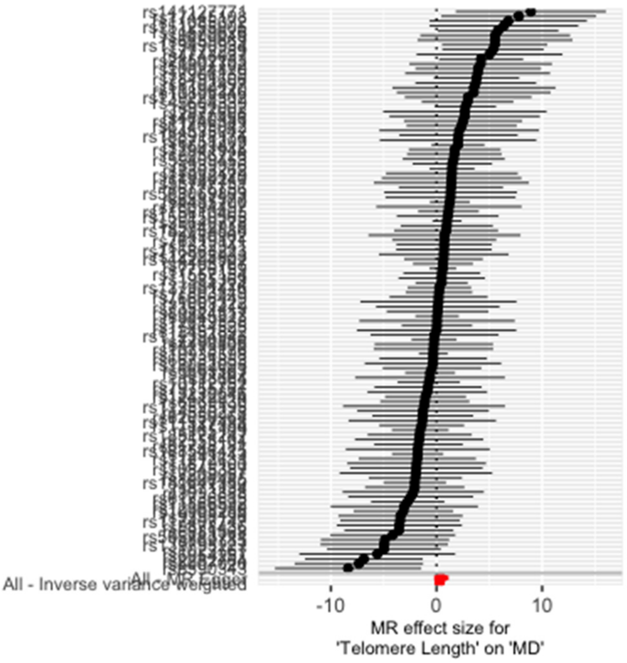


(b) Leave-one-out analysis

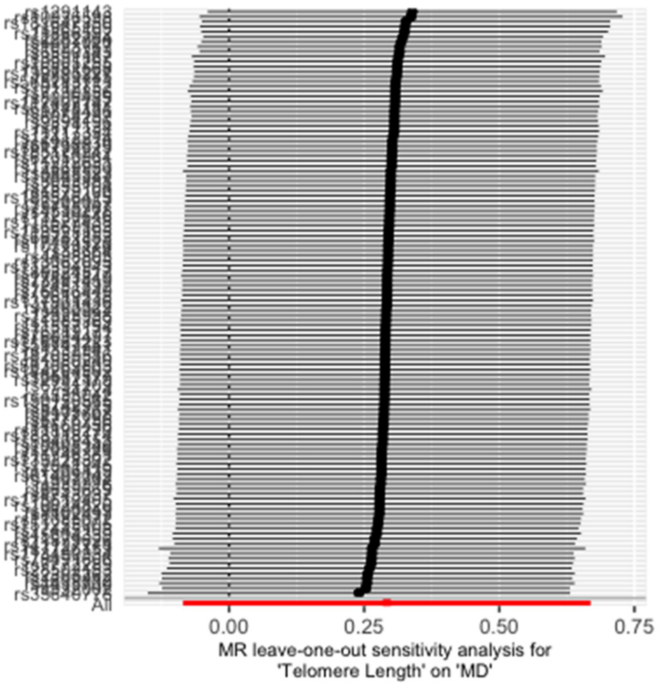


Supplement 2 Figure 4. The single SNP analysis and leave-one-out analysis for LTL on lacunar stroke

(a) Forest plot of single SNP MR

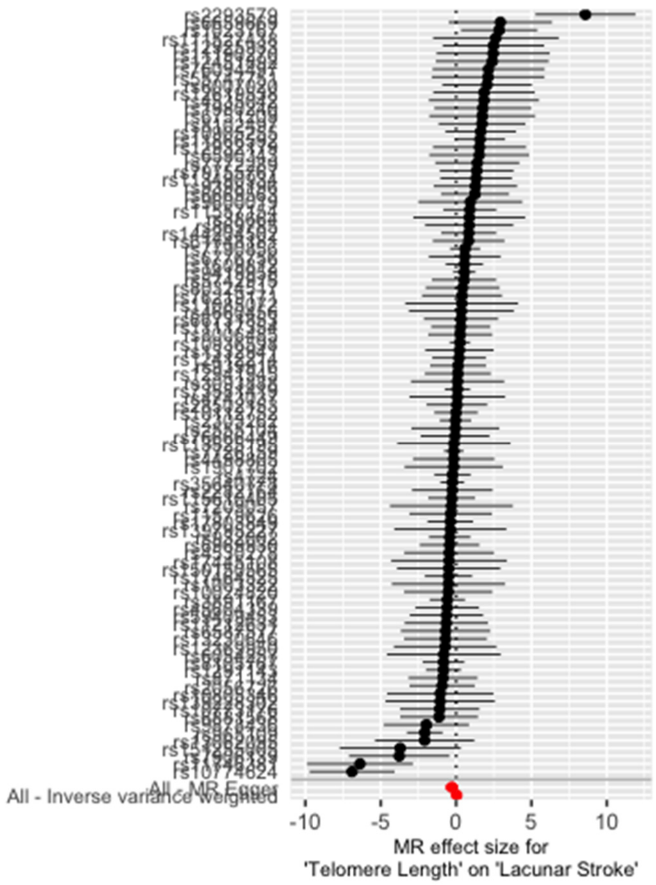


(b) Leave-one-out analysis

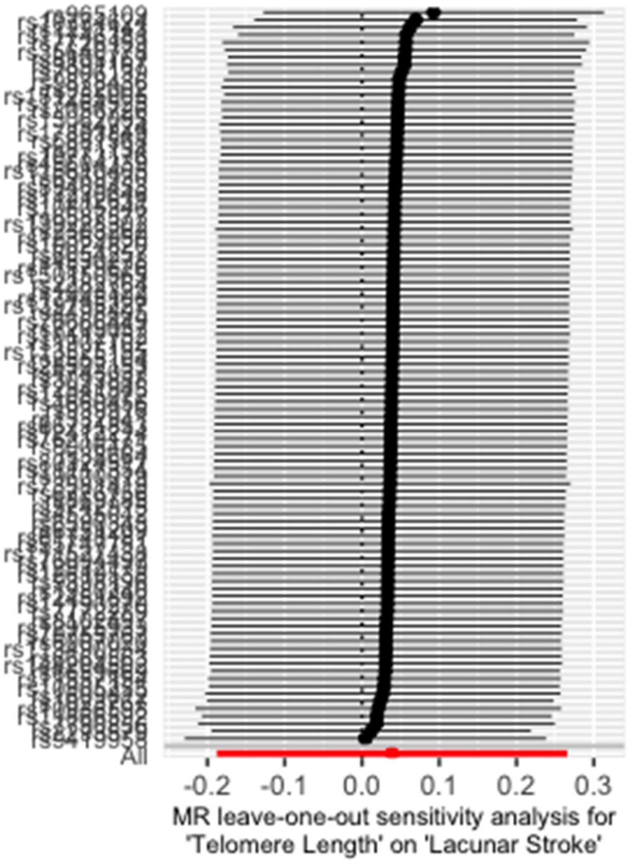

Supplement 2 Figure 5. The single SNP analysis and leave-one-out analysis for LTL on all location BMB

(a) Forest plot of single SNP MR

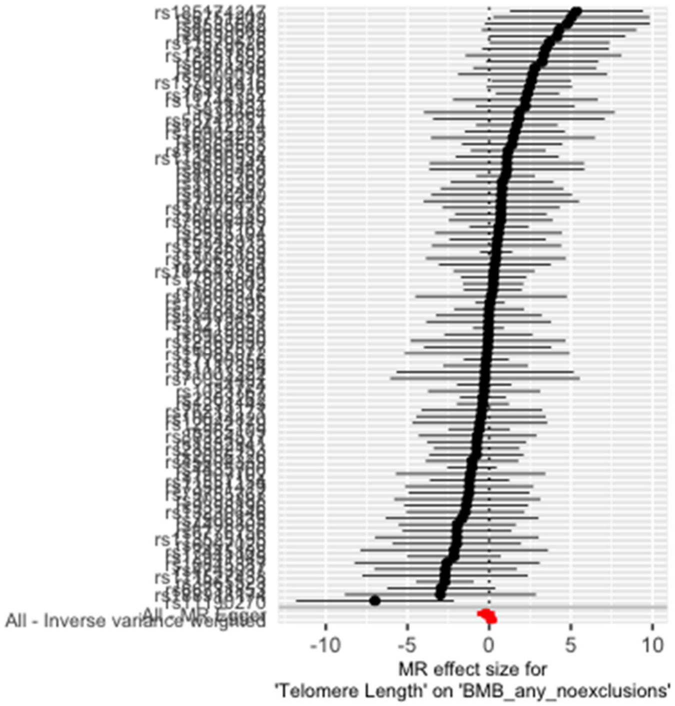


(b) Leave-one-out analysis

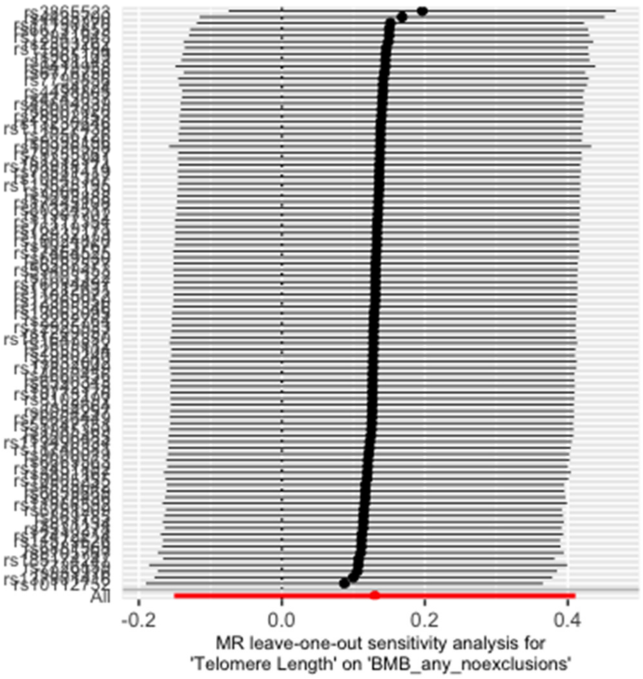


Supplement 2 Figure 6. The single SNP analysis and leave-one-out analysis for LTL on lobar BMB

(a) Forest plot of single SNP MR

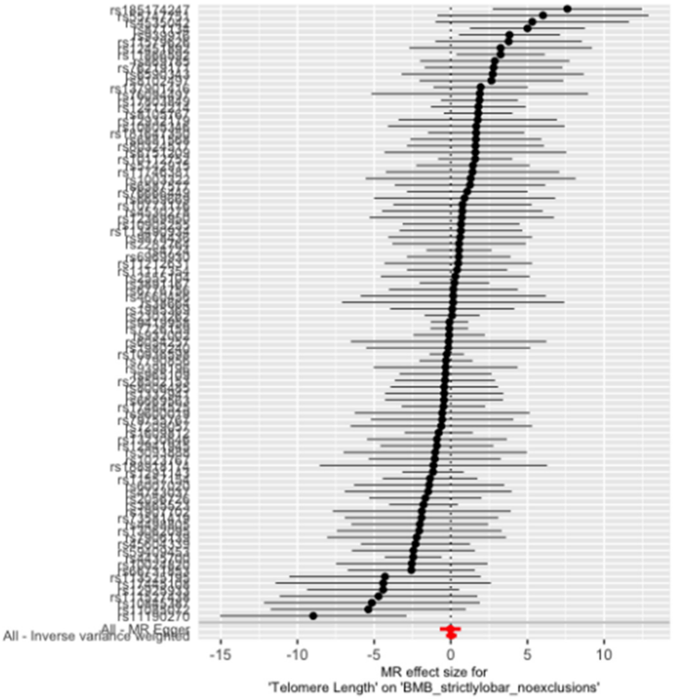


(b) Leave-one-out analysis

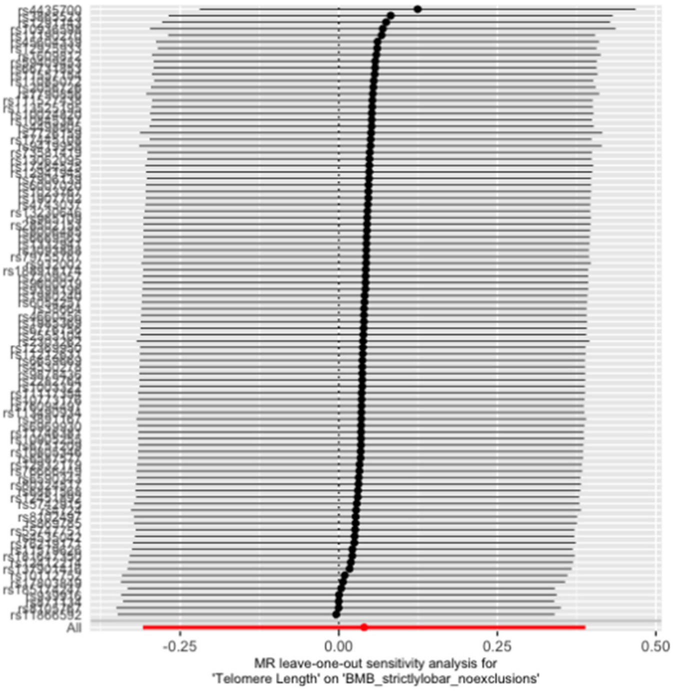


Supplement 2 Figure 7. The single SNP analysis and leave-one-out analysis for LTL on mixed or deep BMB

(a) Forest plot of single SNP MR

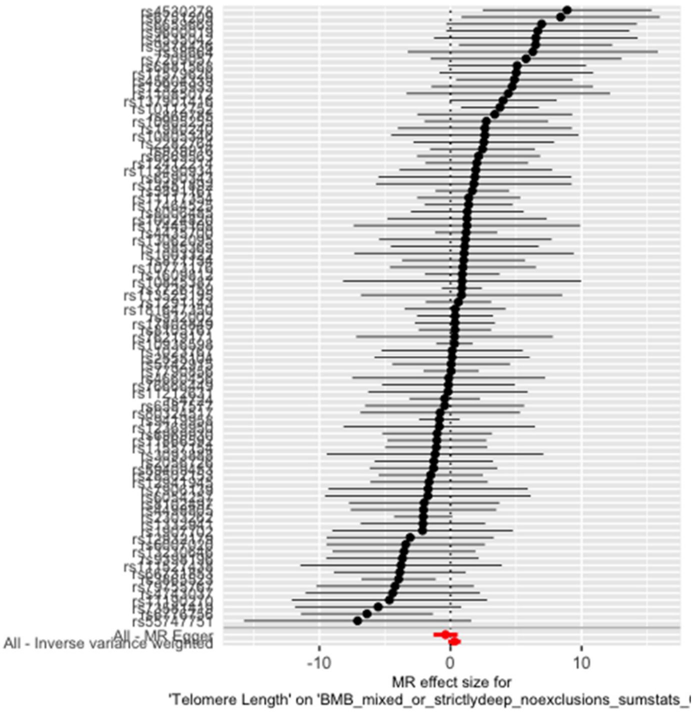


(b) Leave-one-out analysis

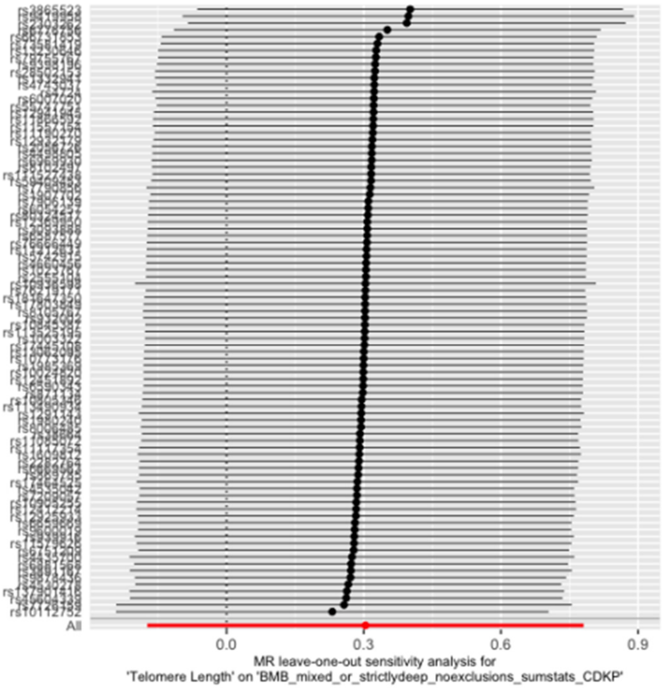


Supplement 2 Figure 8. The single SNP analysis and leave-one-out analysis for LTL on all location ICH or SVS

(a) Forest plot of single SNP MR

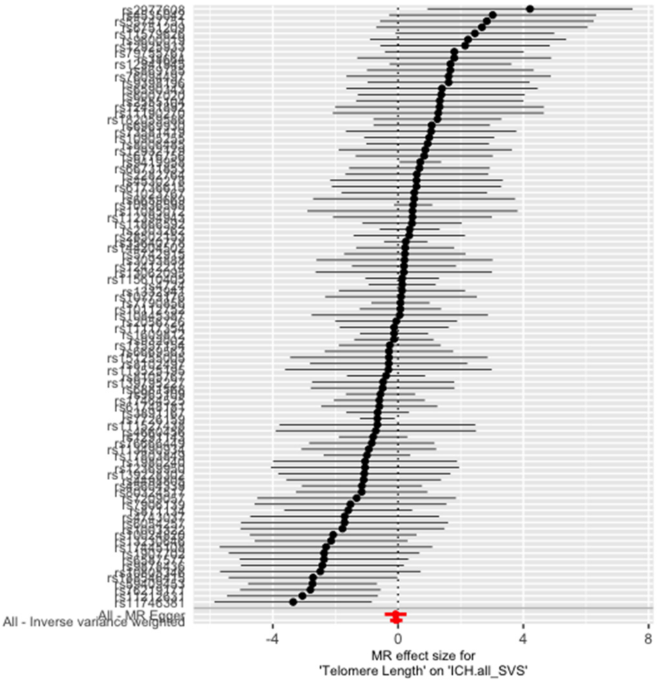


(b) Leave-one-out analysis

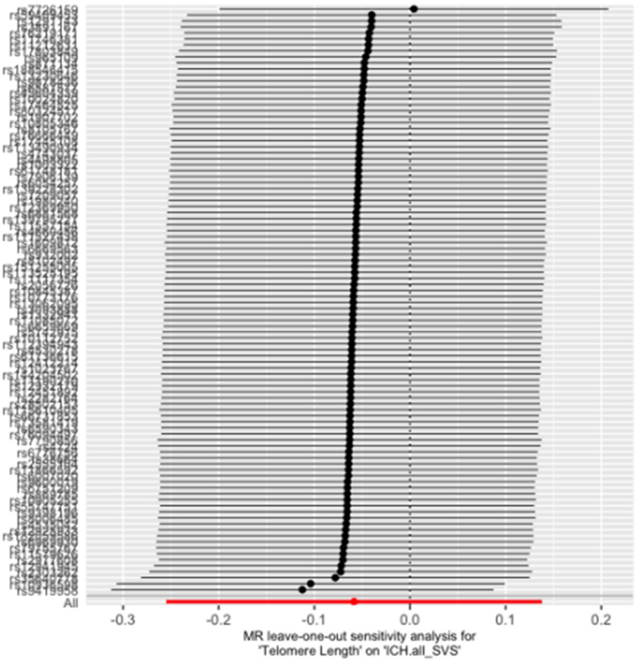


Supplement 2 Figure 9. The single SNP analysis and leave-one-out analysis for LTL on lobar ICH or SVS

(a) Forest plot of single SNP MR

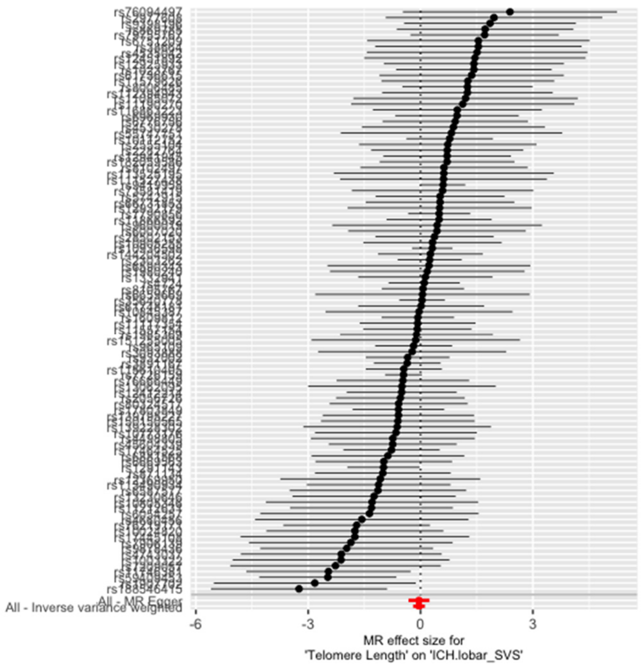


(b) Leave-one-out analysis

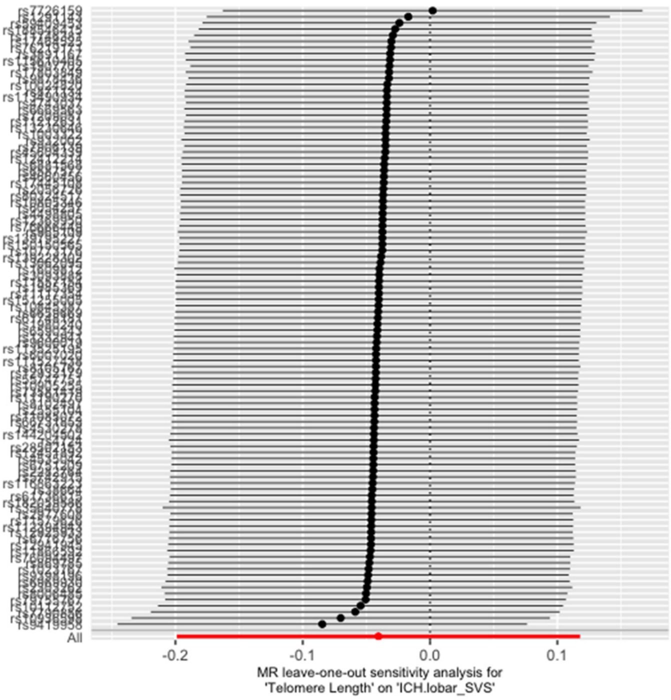


Supplement 2 Figure 10. The single SNP analysis and leave-one-out analysis for LTL on non-lobar ICH or SVS

(a) Forest plot of single SNP MR

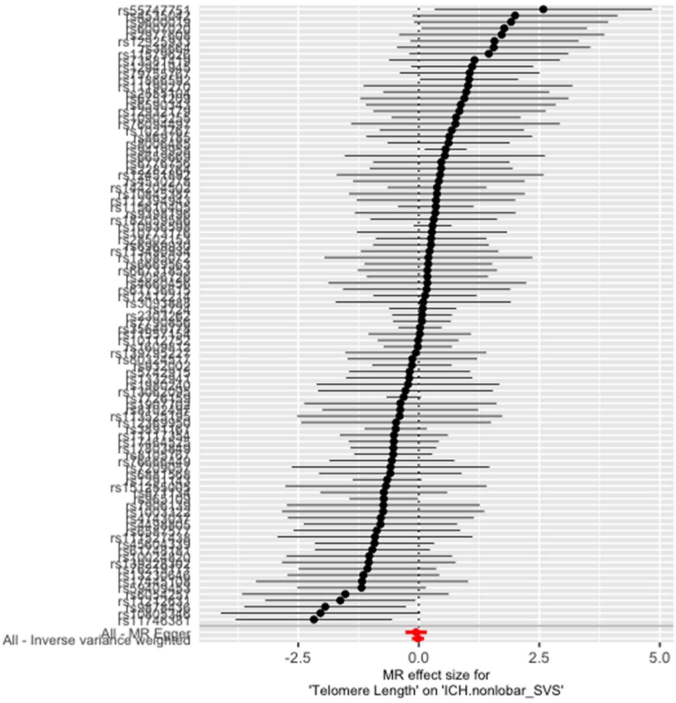


(b) Leave-one-out analysis

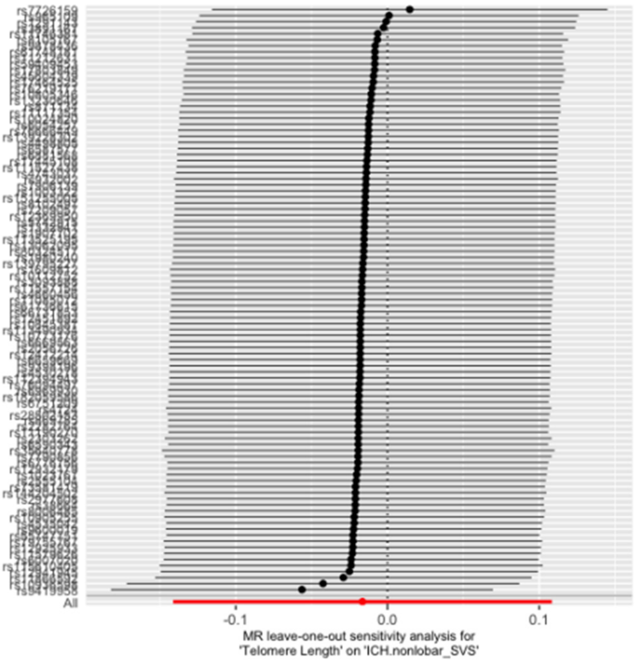


Supplement 2 Figure 11. The single SNP analysis and leave-one-out analysis for GrimAge on WMH volume

(a) Forest plot of single SNP MR

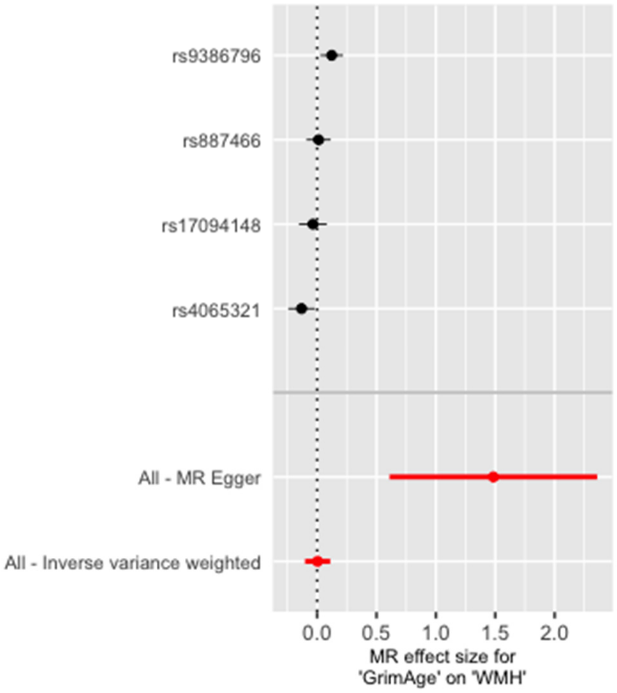


(b) Leave-one-out analysis

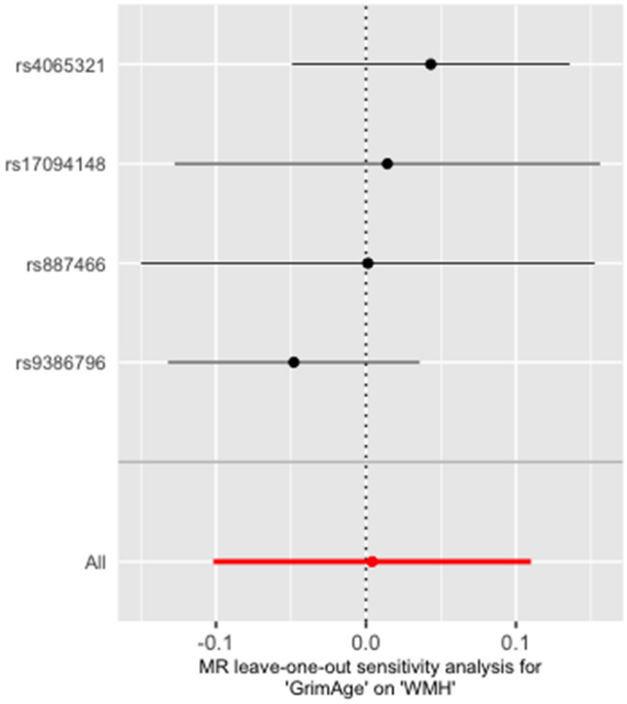


Supplement 2 Figure 12. The single SNP analysis and leave-one-out analysis for GrimAge on FA

(a) Forest plot of single SNP MR

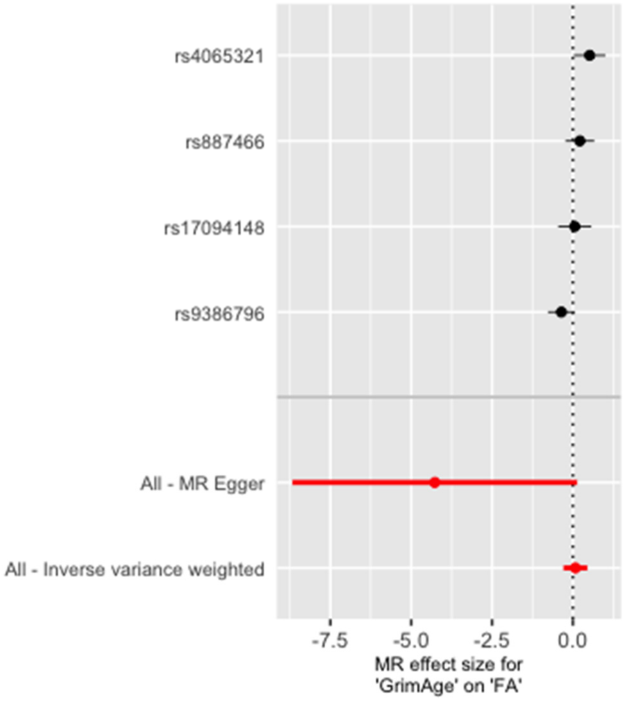


(b) Leave-one-out analysis

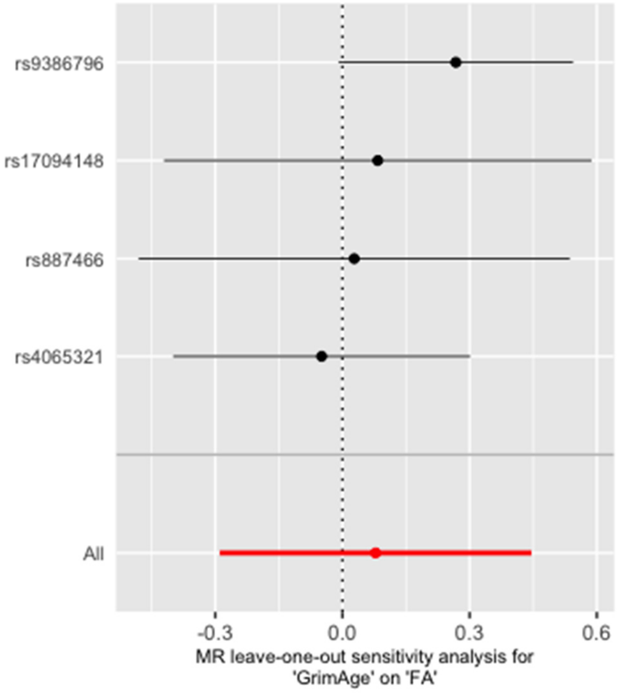


Supplement 2 Figure 13. The single SNP analysis and leave-one-out analysis for GrimAge on MD

(a) Forest plot of single SNP MR

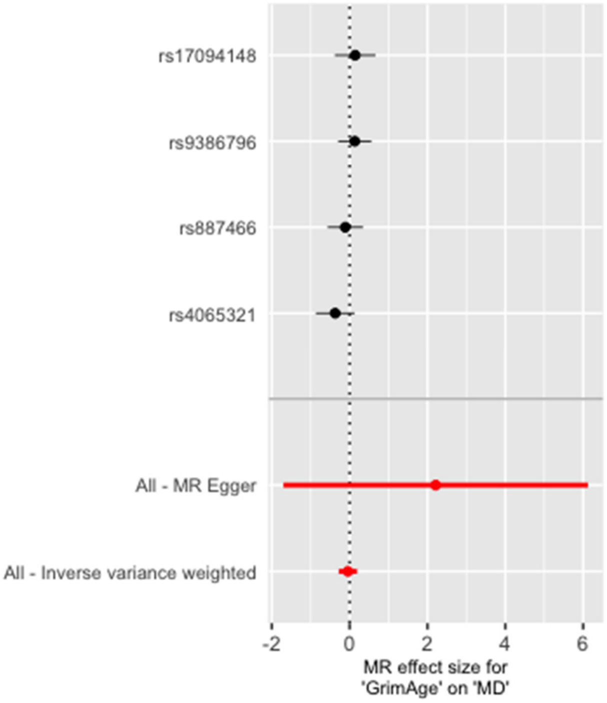


(b) Leave-one-out analysis

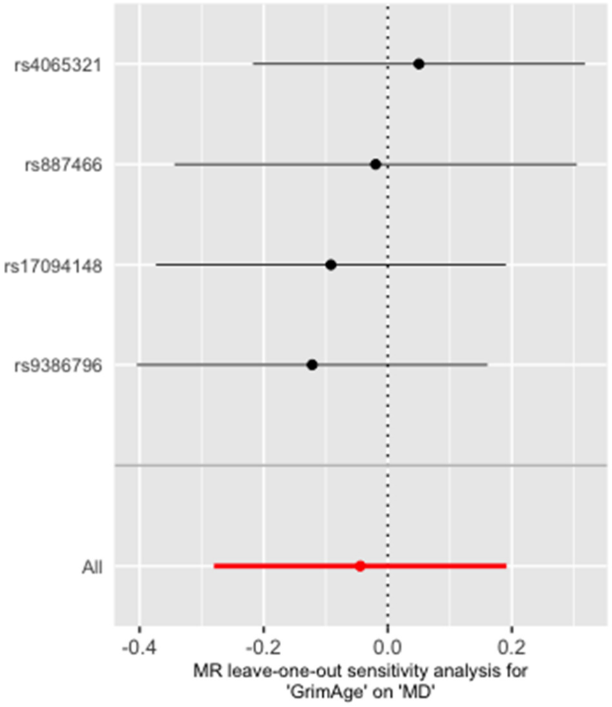


Supplement 2 Figure 14. The single SNP analysis and leave-one-out analysis for GrimAge on lacunar stroke

(a) Forest plot of single SNP MR

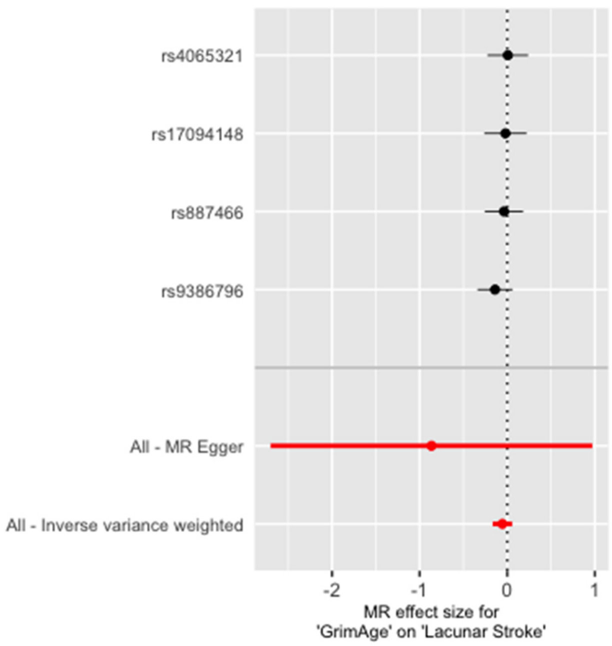


(b) Leave-one-out analysis

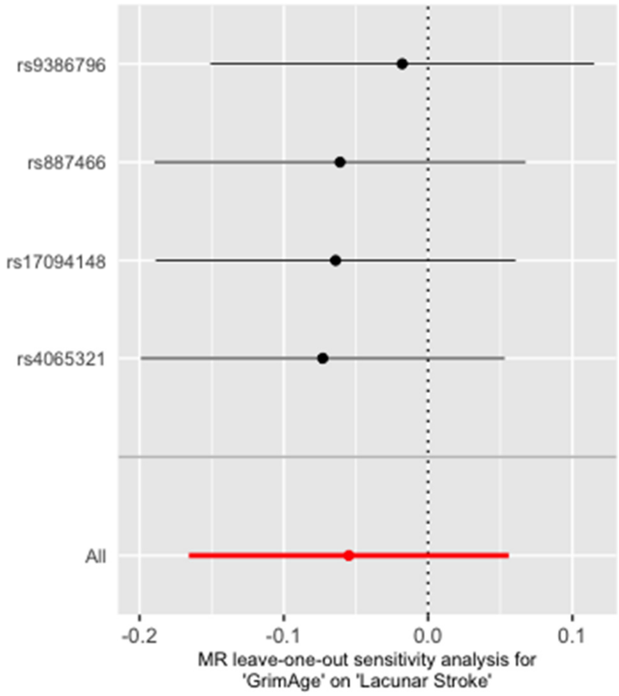


Supplement 2 Figure 15. The single SNP analysis and leave-one-out analysis for GrimAge on all location BMB

(a) Forest plot of single SNP MR

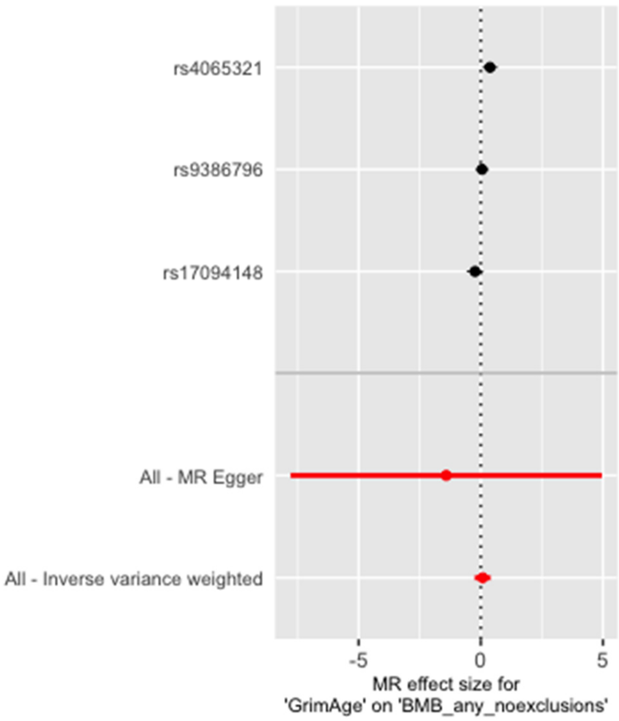


(b) Leave-one-out analysis

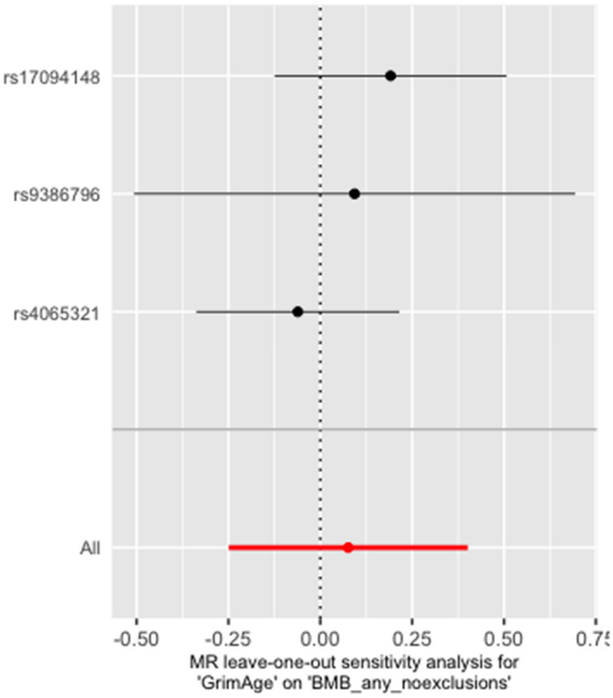


Supplement 2 Figure 16. The single SNP analysis and leave-one-out analysis for GrimAge on lobar BMB

(a) Forest plot of single SNP MR

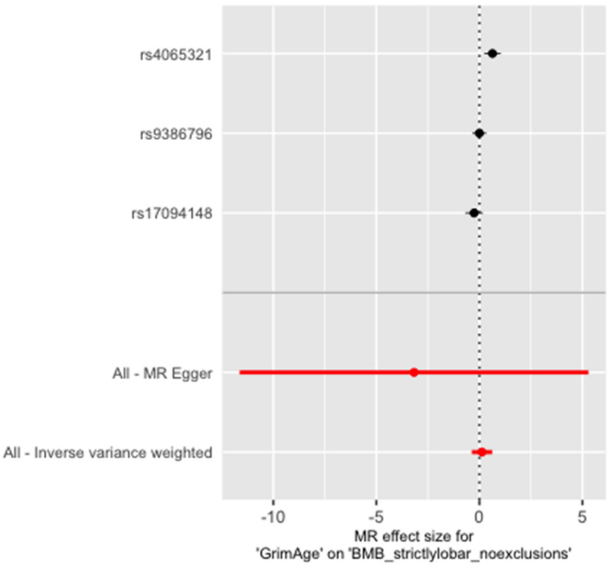


(b) Leave-one-out analysis

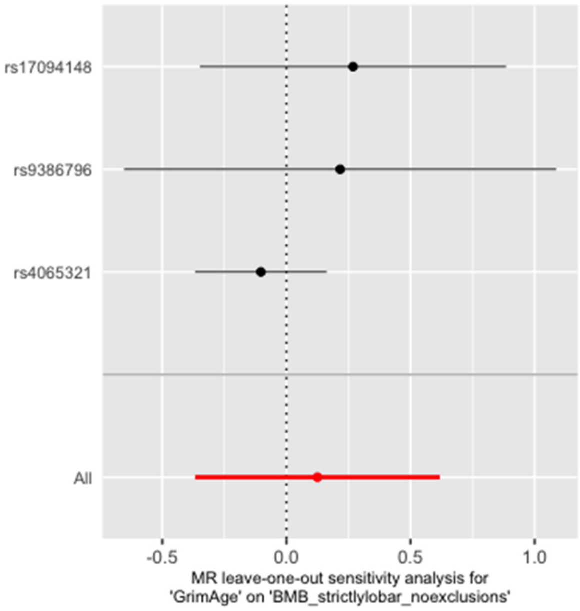


Supplement 2 Figure 17. The single SNP analysis and leave-one-out analysis for GrimAge on mixed or deep BMB

(a) Forest plot of single SNP MR

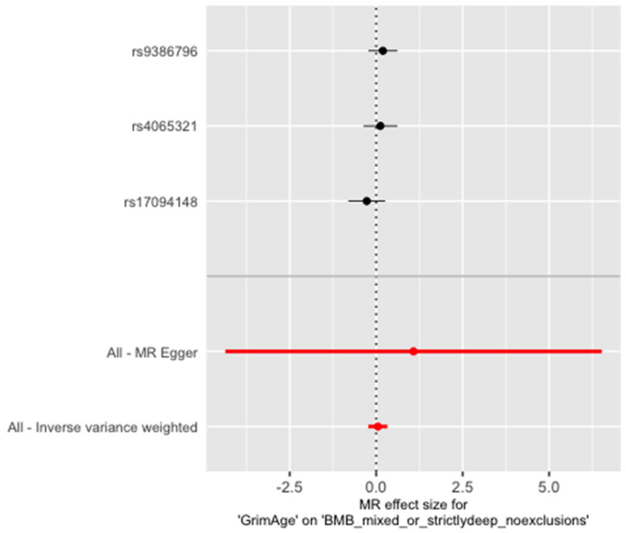


(b) Leave-one-out analysis

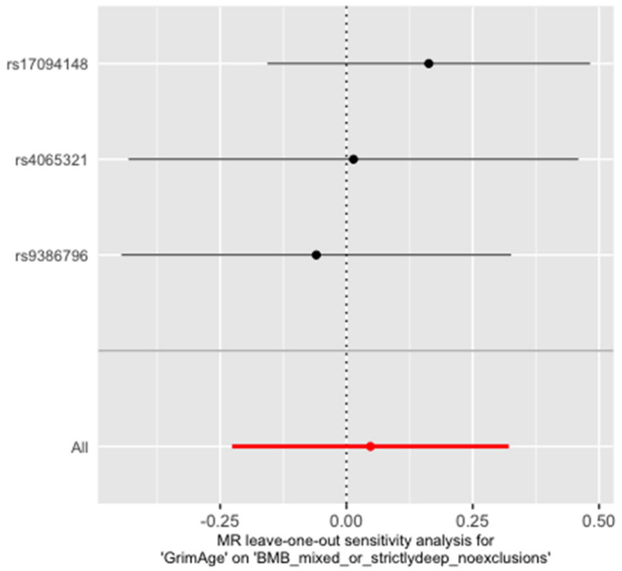


Supplement 2 Figure 18. The single SNP analysis and leave-one-out analysis for GrimAge on all location ICH or SVS

(a) Forest plot of single SNP MR

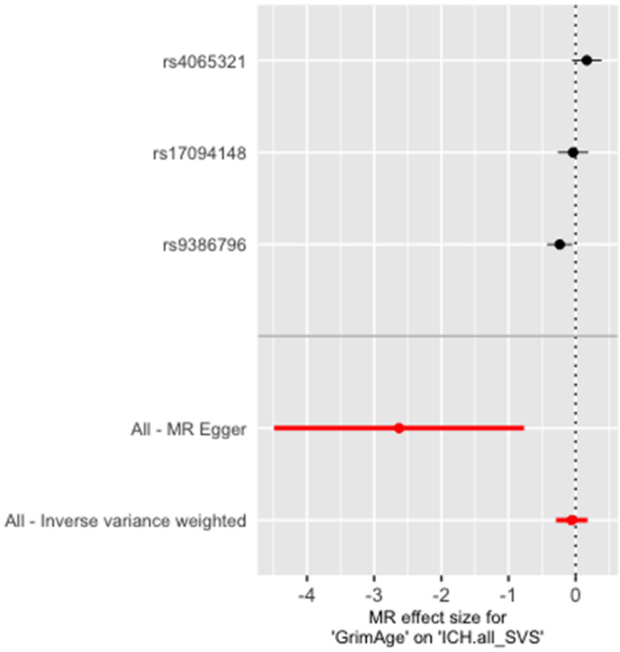


(b) Leave-one-out analysis

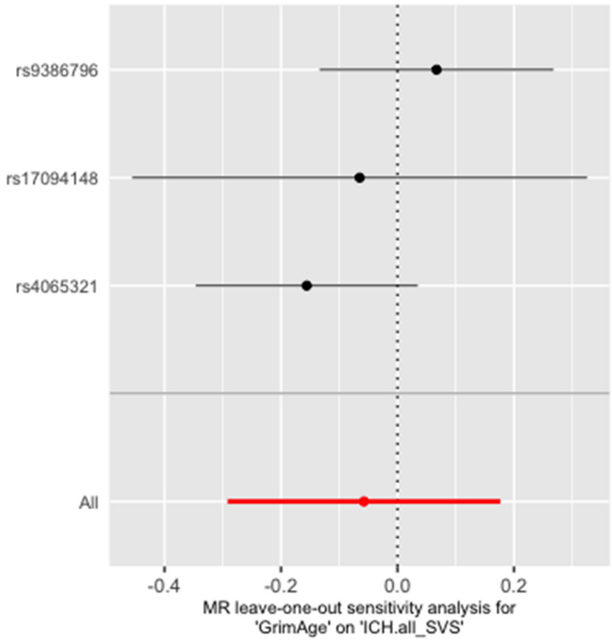


Supplement 2 Figure 19. The single SNP analysis and leave-one-out analysis for GrimAge on lobar ICH or SVS

(a) Forest plot of single SNP MR

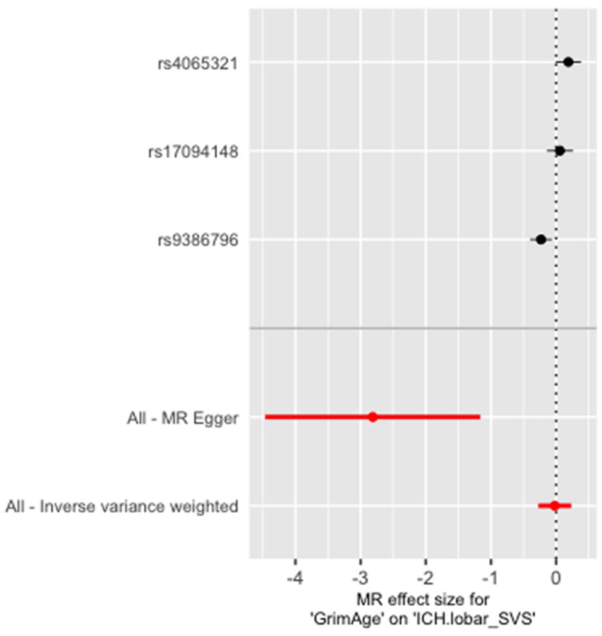


(b) Leave-one-out analysis

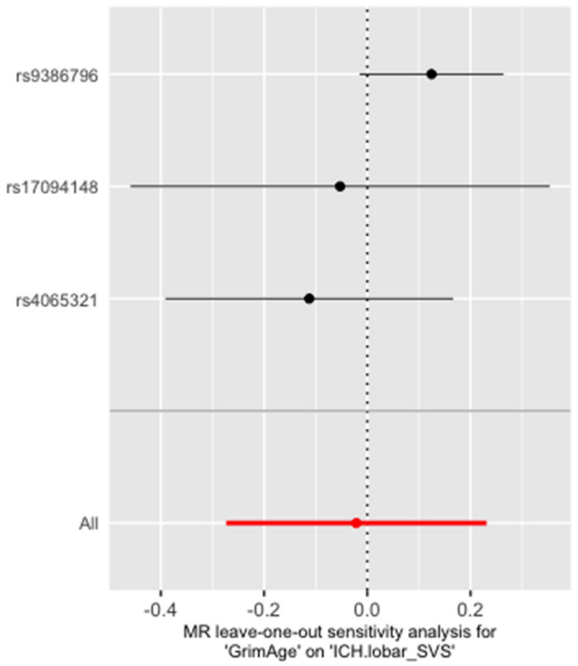


Supplement 2 Figure 20. The single SNP analysis and leave-one-out analysis for GrimAge on non-lobar ICH or SVS

(a) Forest plot of single SNP MR

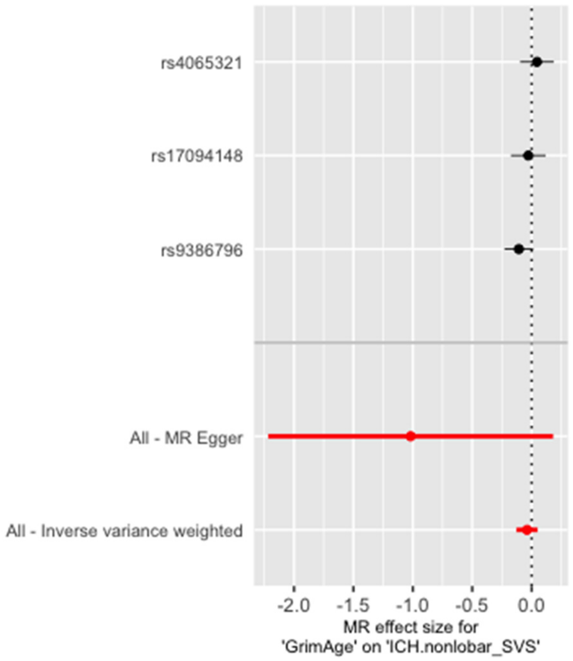


(b) Leave-one-out analysis

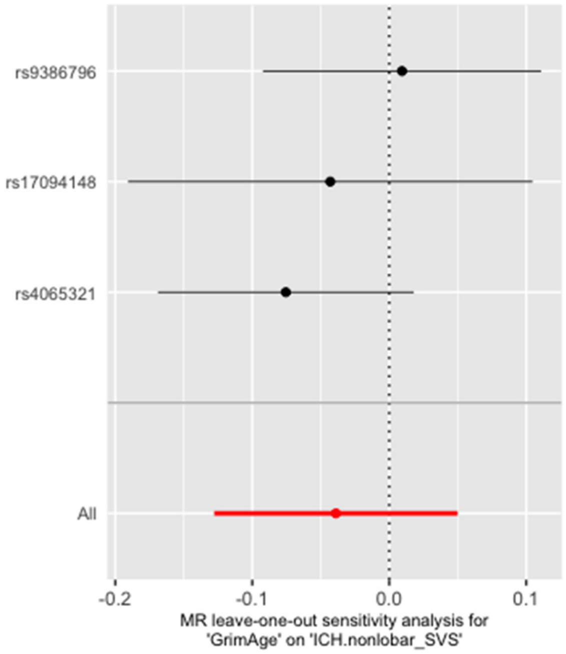


Supplement 2 Figure 21. The single SNP analysis and leave-one-out analysis for PhenoAge on WMH volume

(a) Forest plot of single SNP MR

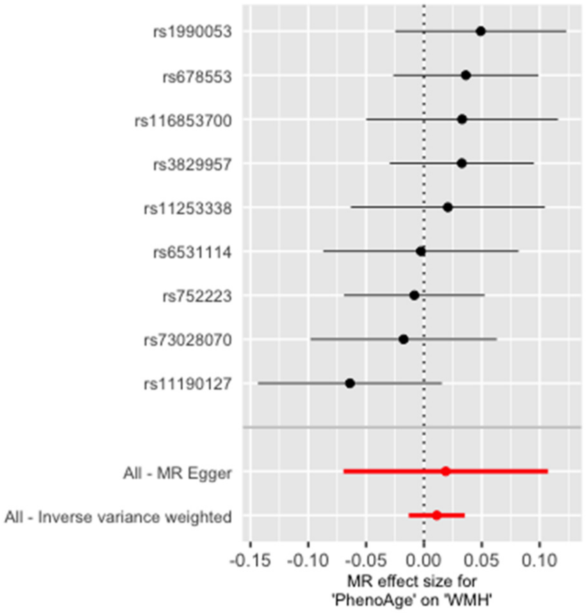


(b) Leave-one-out analysis

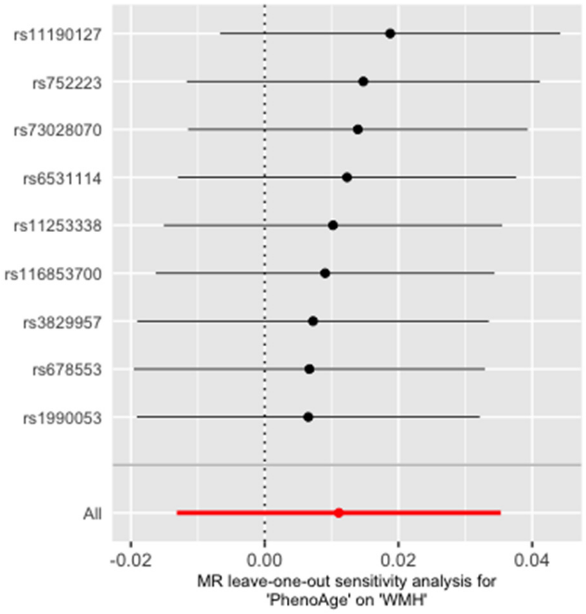


Supplement 2 Figure 22. The single SNP analysis and leave-one-out analysis for PhenoAge on FA

(a) Forest plot of single SNP MR

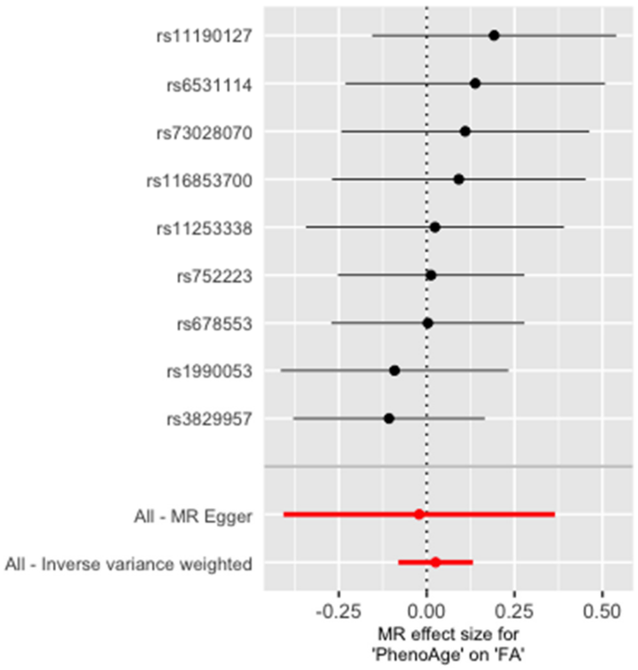


(b) Leave-one-out analysis

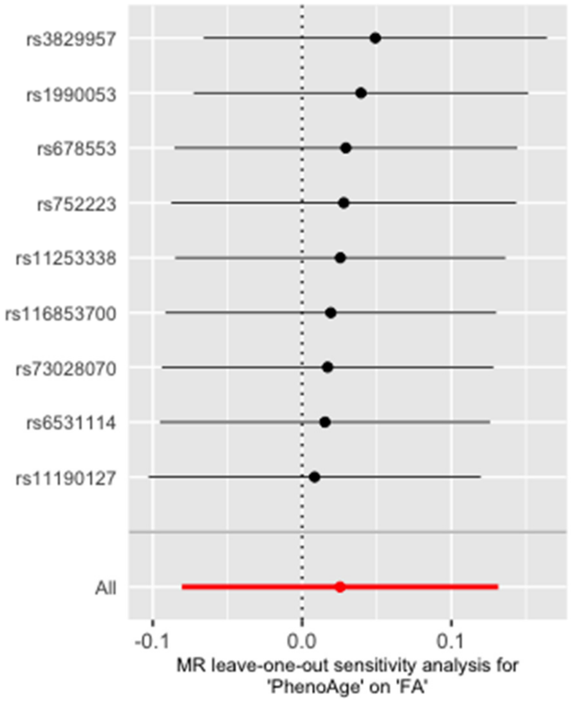


Supplement 2 Figure 23. The single SNP analysis and leave-one-out analysis for PhenoAge on MD

(a) Forest plot of single SNP MR

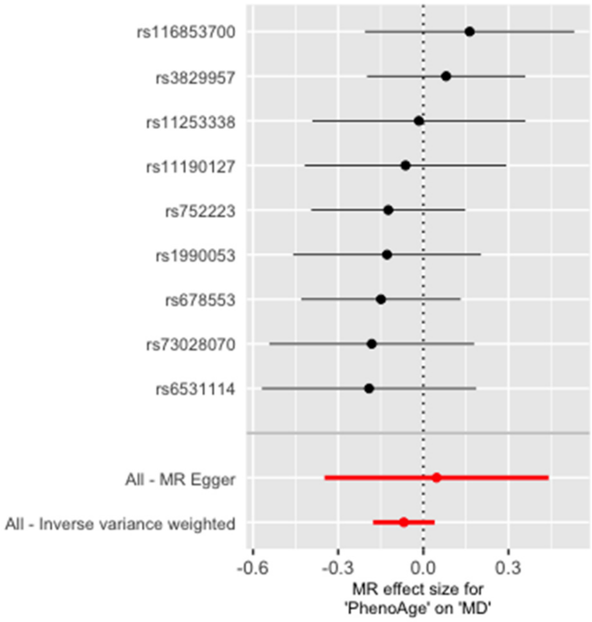


(b) Leave-one-out analysis

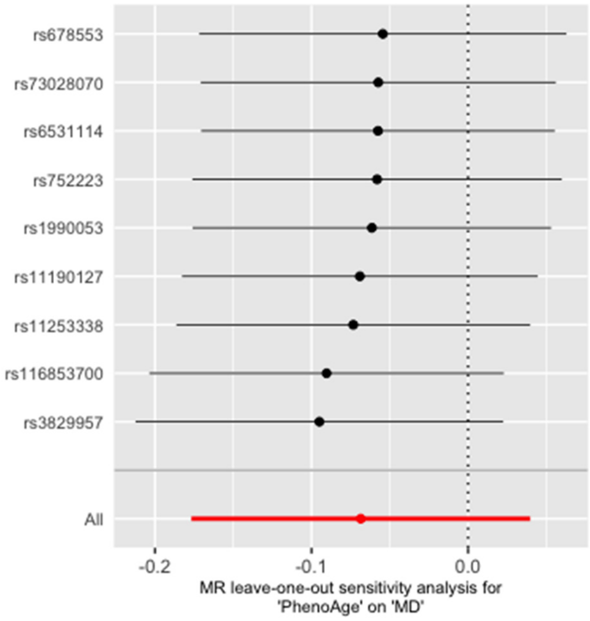


Supplement 2 Figure 24. The single SNP analysis and leave-one-out analysis for PhenoAge on lacunar stroke

(a) Forest plot of single SNP MR

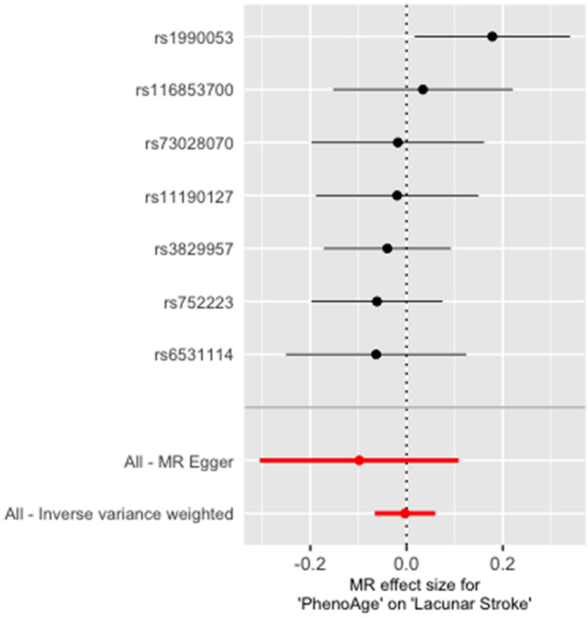


(b) Leave-one-out analysis

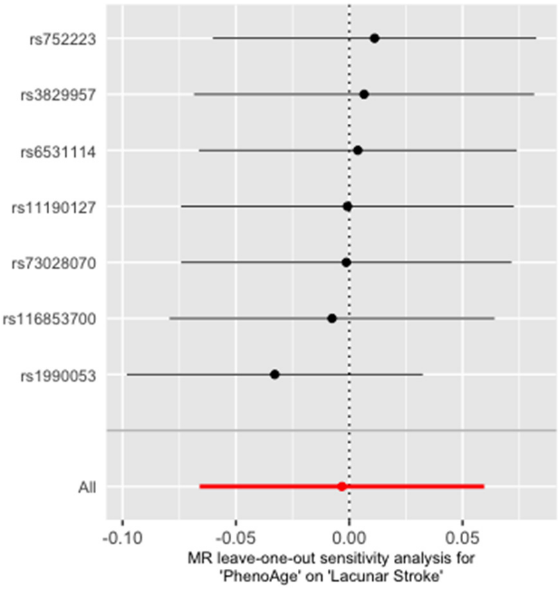


Supplement 2 Figure 25. The single SNP analysis and leave-one-out analysis for PhenoAge on all location BMB

(a) Forest plot of single SNP MR

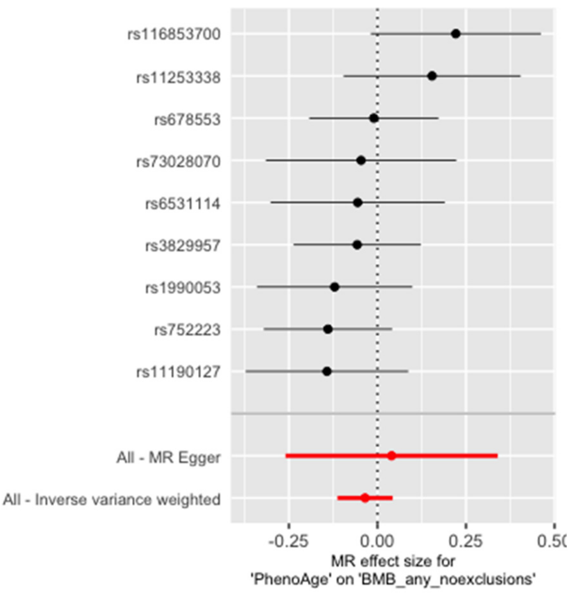


(b) Leave-one-out analysis

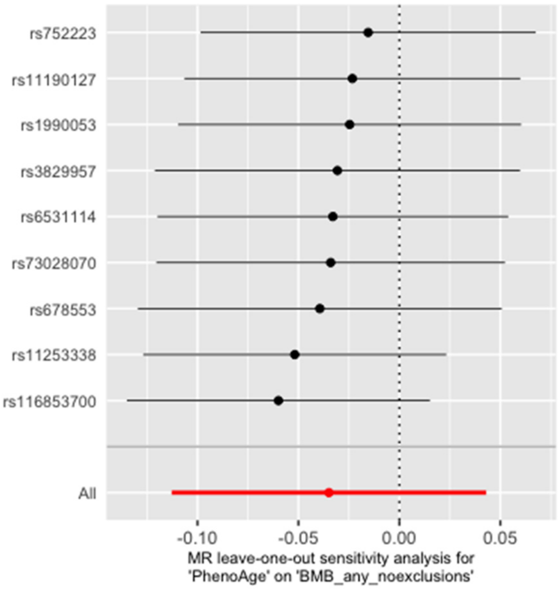


Supplement 2 Figure 26. The single SNP analysis and leave-one-out analysis for PhenoAge on lobar BMB

(a) Forest plot of single SNP MR

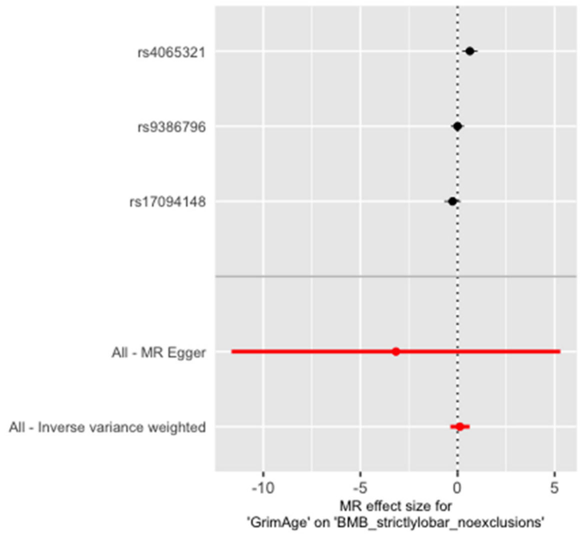


(b) Leave-one-out analysis

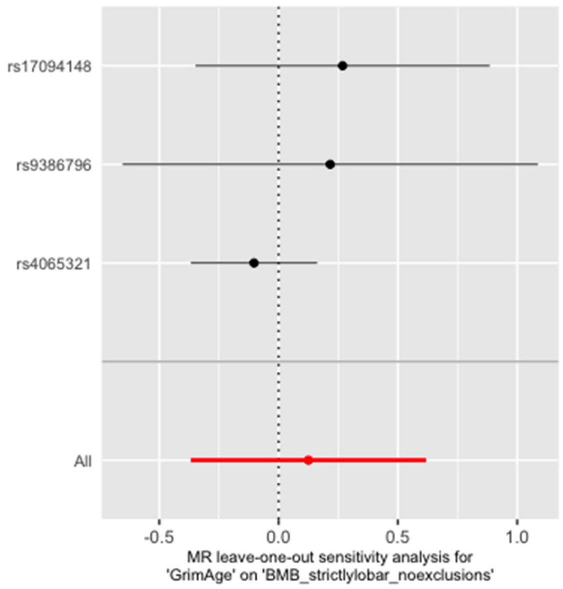


Supplement 2 Figure 27. The single SNP analysis and leave-one-out analysis for PhenoAge on mixed or deep BMB

(a) Forest plot of single SNP MR

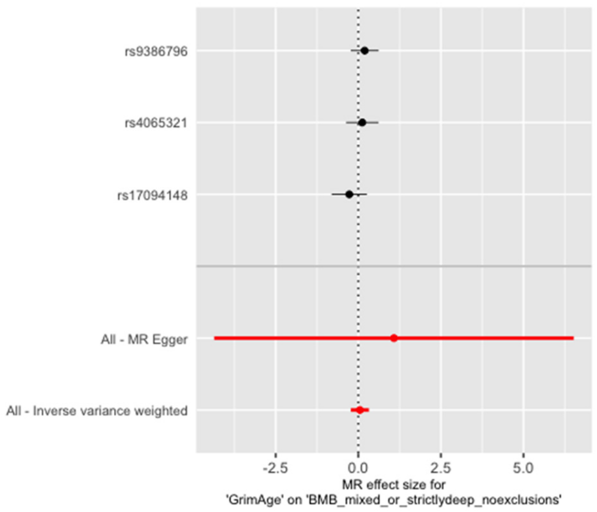


(b) Leave-one-out analysis

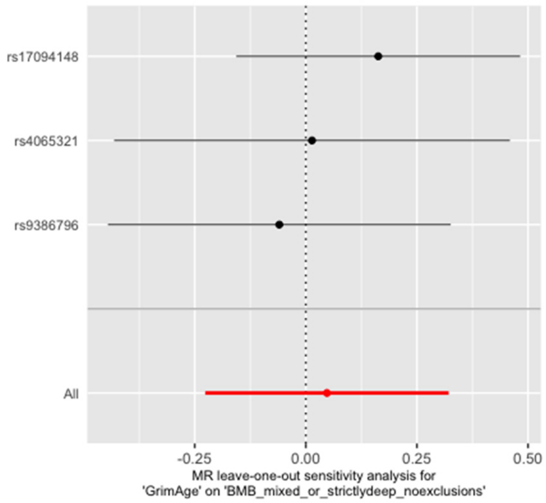


Supplement 2 Figure 28. The single SNP analysis and leave-one-out analysis for PhenoAge on all location ICH or SVS

(a) Forest plot of single SNP MR

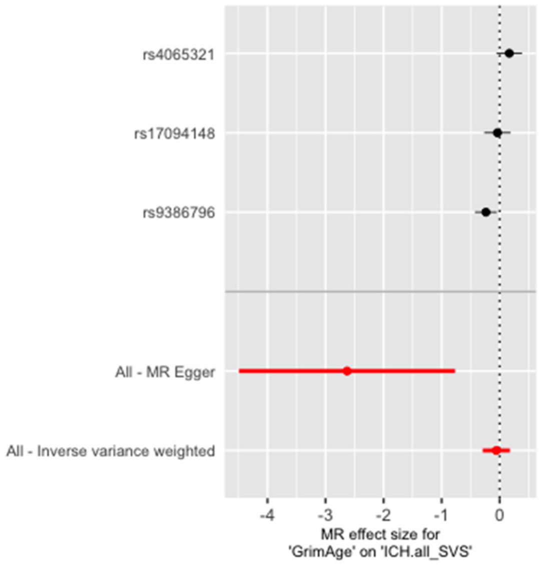


(b) Leave-one-out analysis

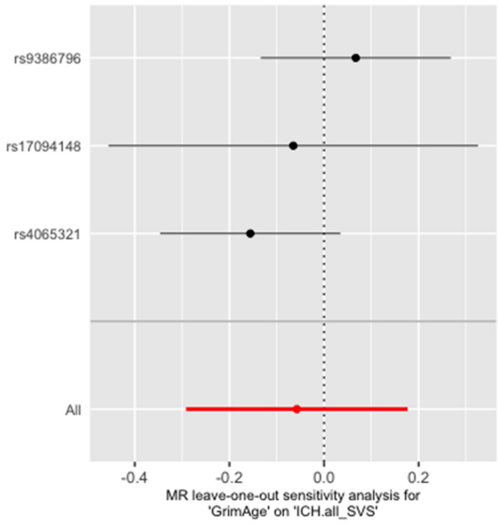


Supplement 2 Figure 29. The single SNP analysis and leave-one-out analysis for PhenoAge on lobar ICH or SVS

(a) Forest plot of single SNP MR

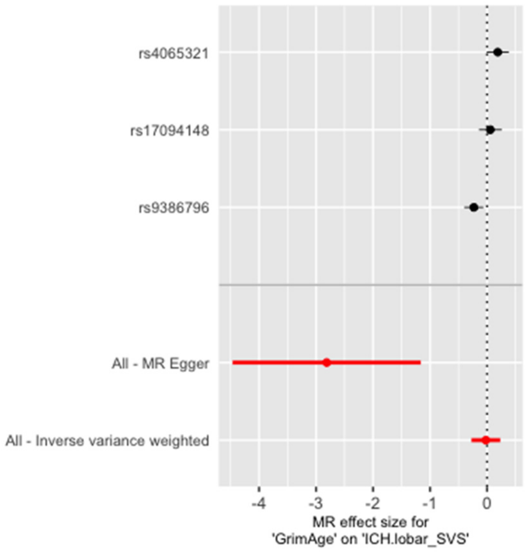


(b) Leave-one-out analysis

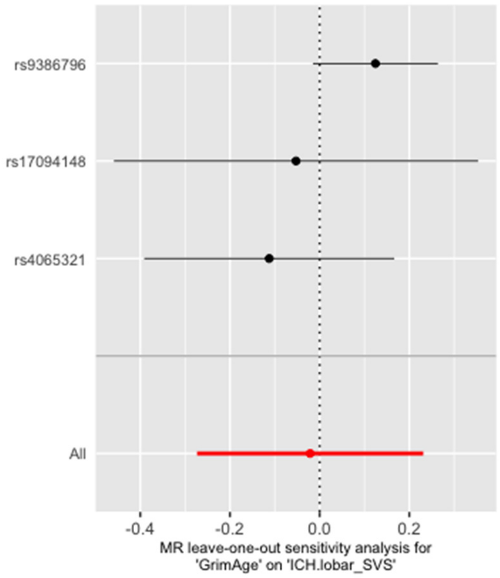


Supplement 2 Figure 30. The single SNP analysis and leave-one-out analysis for PhenoAge on non-lobar ICH or SVS

(a) Forest plot of single SNP MR

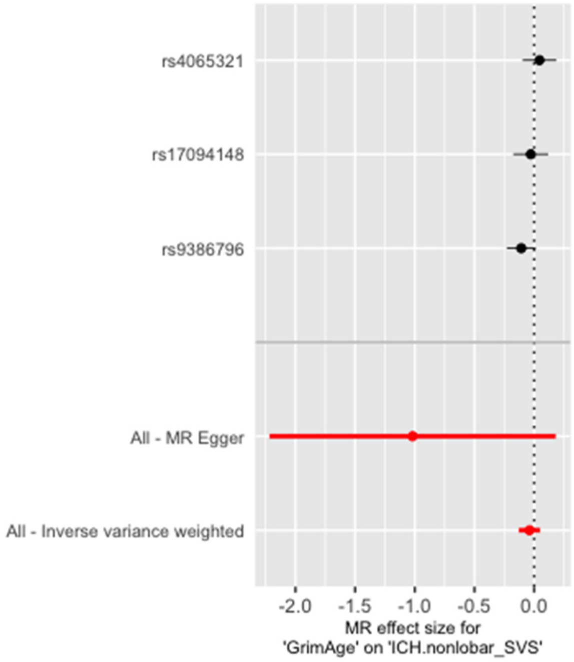


(b) Leave-one-out analysis

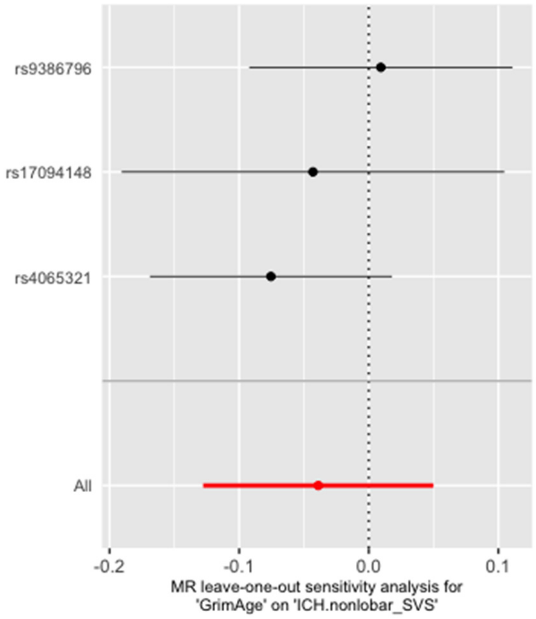


Supplement 2 Figure 31. The single SNP analysis and leave-one-out analysis for HannumAge on WMH volume

(a) Forest plot of single SNP MR

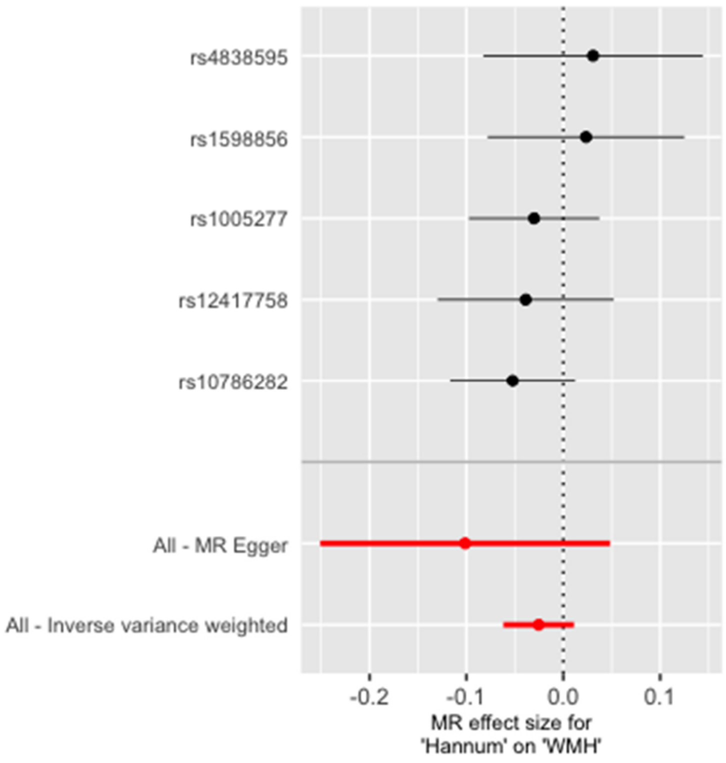


(b) Leave-one-out analysis

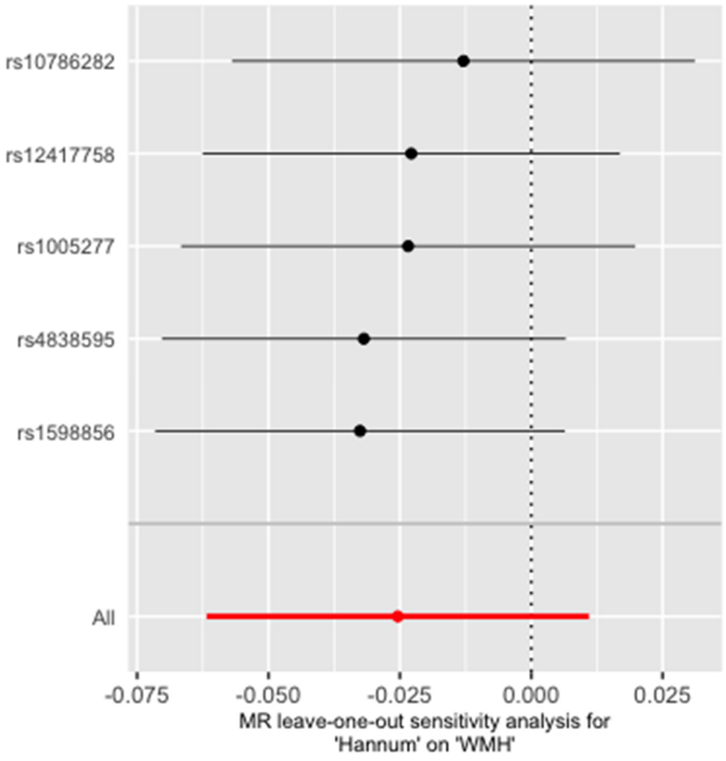


Supplement 2 Figure 32. The single SNP analysis and leave-one-out analysis for HannumAge on FA

(a) Forest plot of single SNP MR

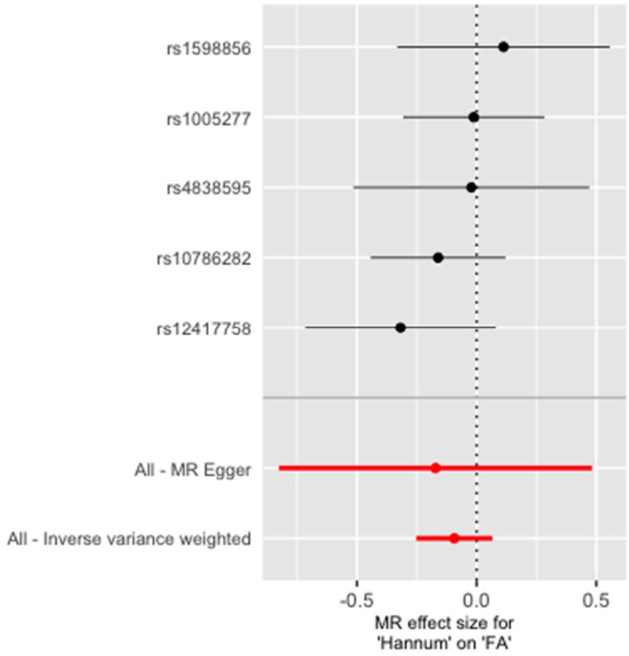


(b) Leave-one-out analysis

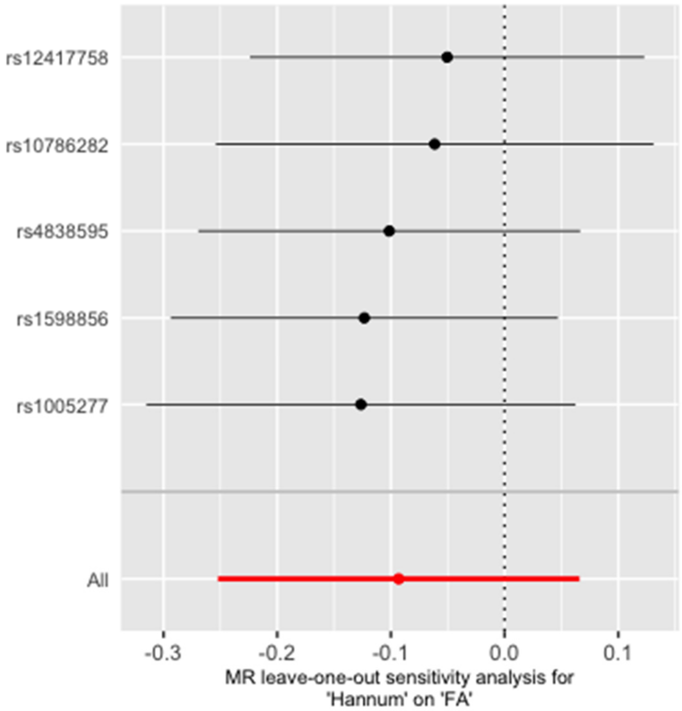


Supplement 2 Figure 33. The single SNP analysis and leave-one-out analysis for HannumAge on MD

(a) Forest plot of single SNP MR

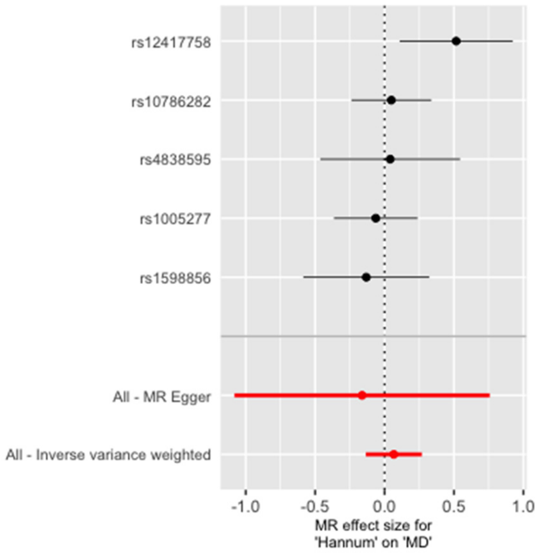


(b) Leave-one-out analysis

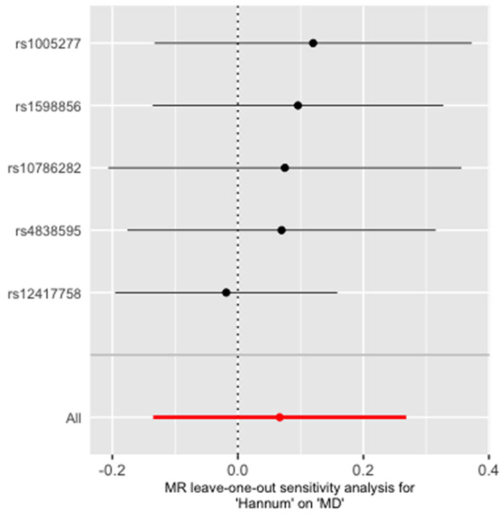


Supplement 2 Figure 34. The single SNP analysis and leave-one-out analysis for HannumAge on lacunar stroke

(a) Forest plot of single SNP MR

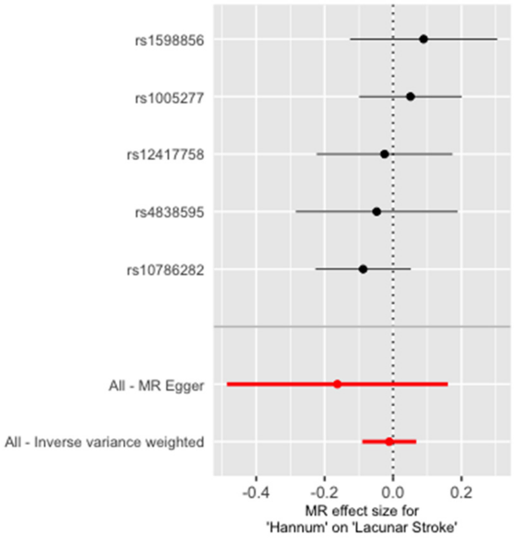

(b) Leave-one-out analysis

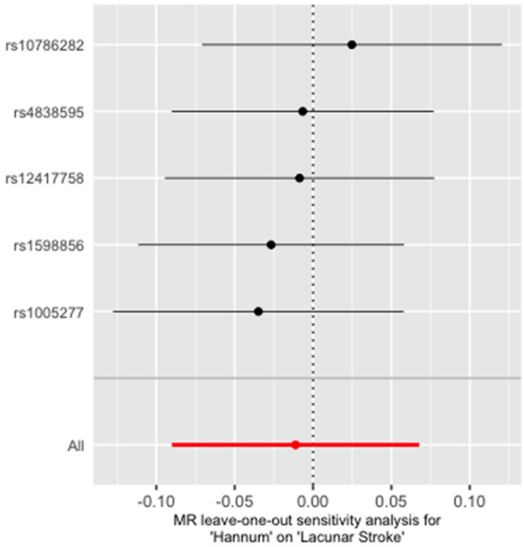


Supplement 2 Figure 35. The single SNP analysis and leave-one-out analysis for HannumAge on all location BMB

(a) Forest plot of single SNP MR

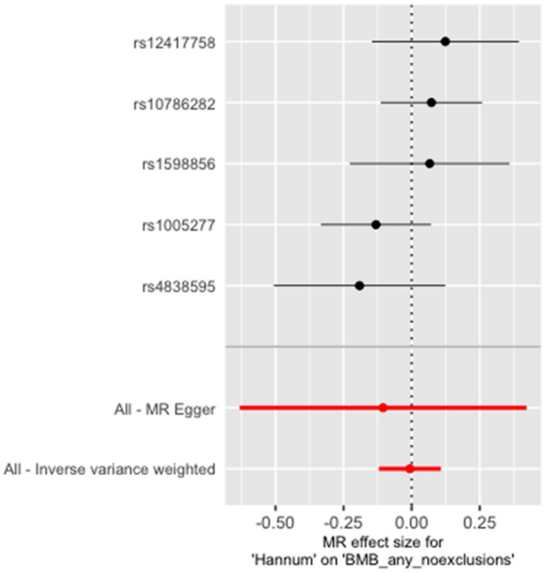


(b) Leave-one-out analysis

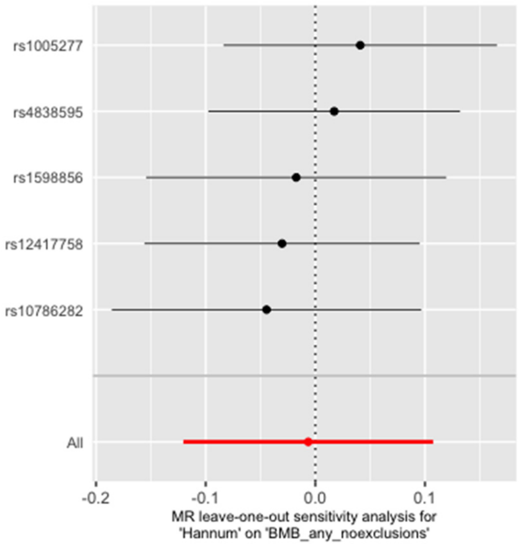


Supplement 2 Figure 36. The single SNP analysis and leave-one-out analysis for HannumAge on lobar BMB

(a) Forest plot of single SNP MR

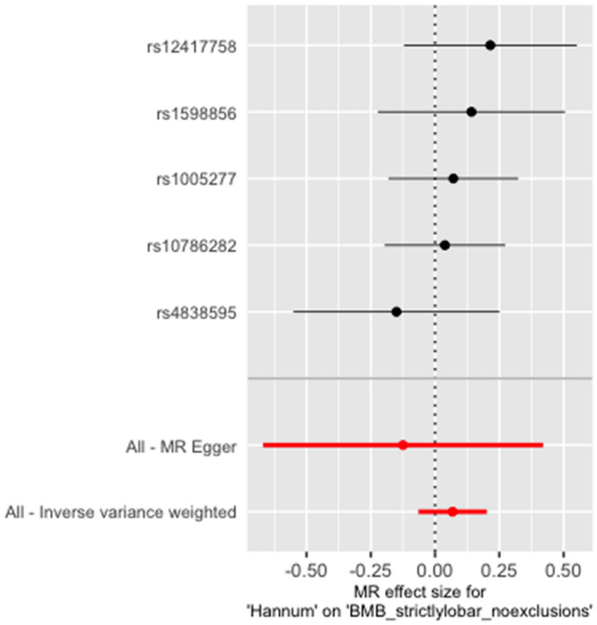


(b) Leave-one-out analysis

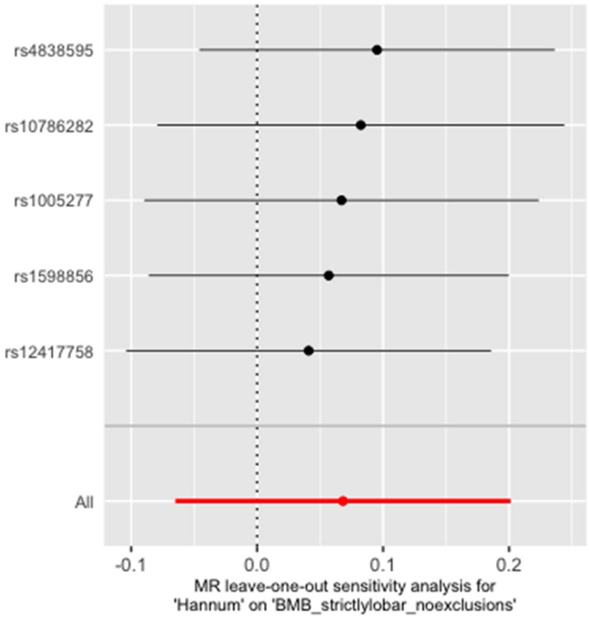


Supplement 2 Figure 37. The single SNP analysis and leave-one-out analysis for HannumAge on mixed or deep BMB

(a) Forest plot of single SNP MR

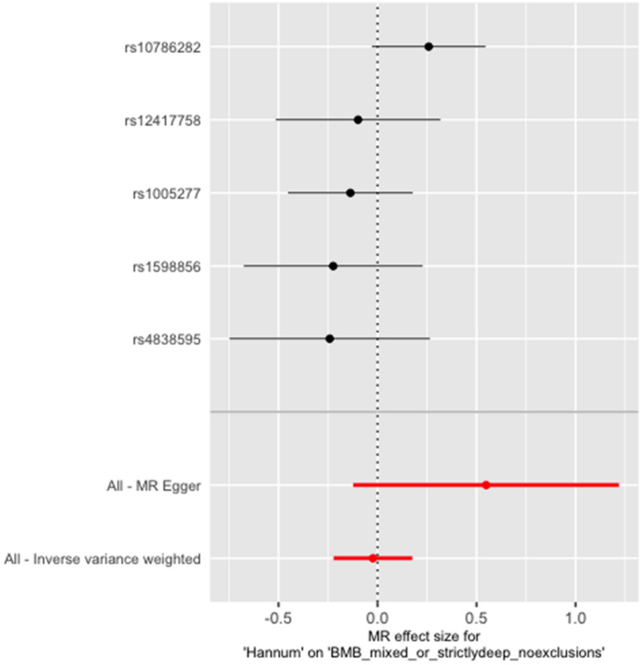


(b) Leave-one-out analysis

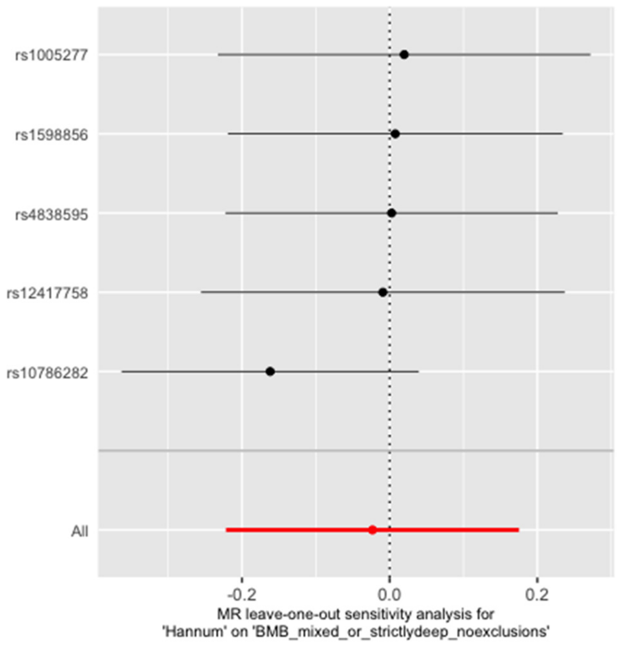


Supplement 2 Figure 38. The single SNP analysis and leave-one-out analysis for HannumAge on all location ICH or SVS

(a) Forest plot of single SNP MR

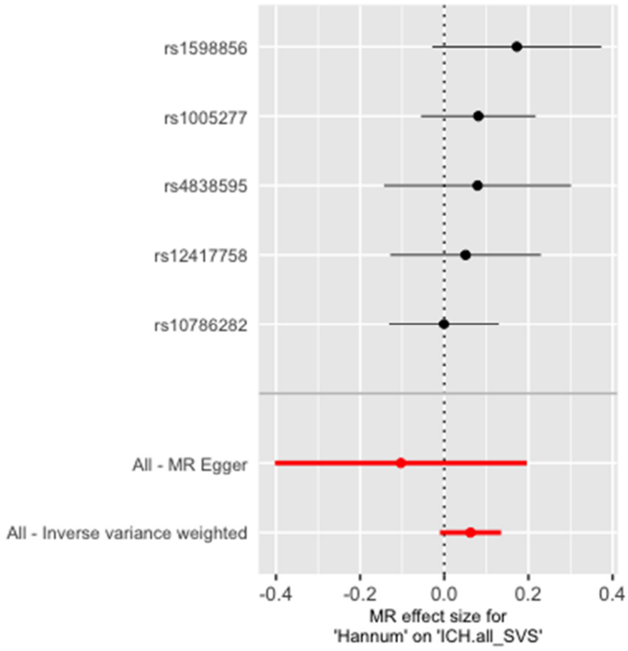


(b) Leave-one-out analysis

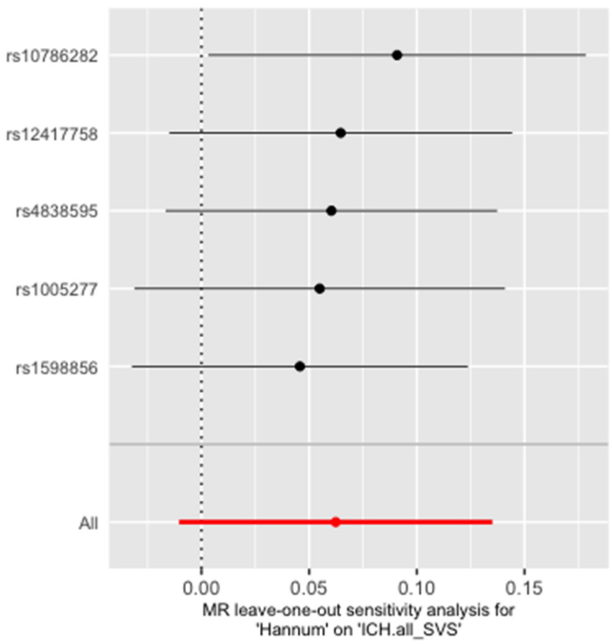


Supplement 2 Figure 39. The single SNP analysis and leave-one-out analysis for HannumAge on lobar ICH or SVS

(a) Forest plot of single SNP MR

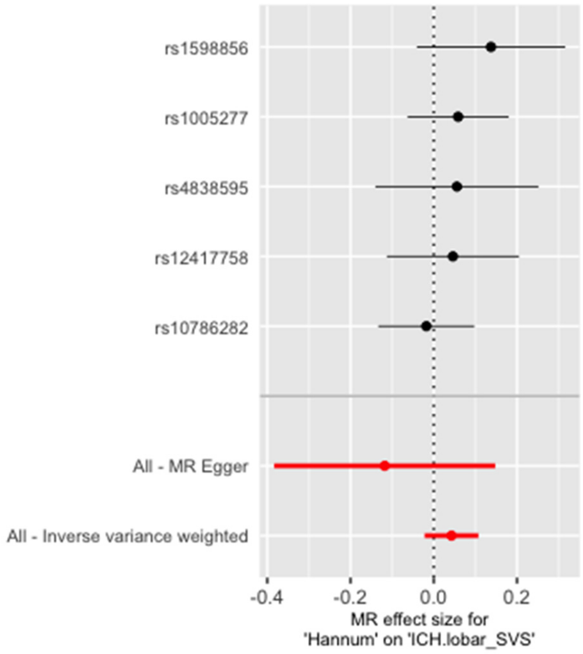


(b) Leave-one-out analysis

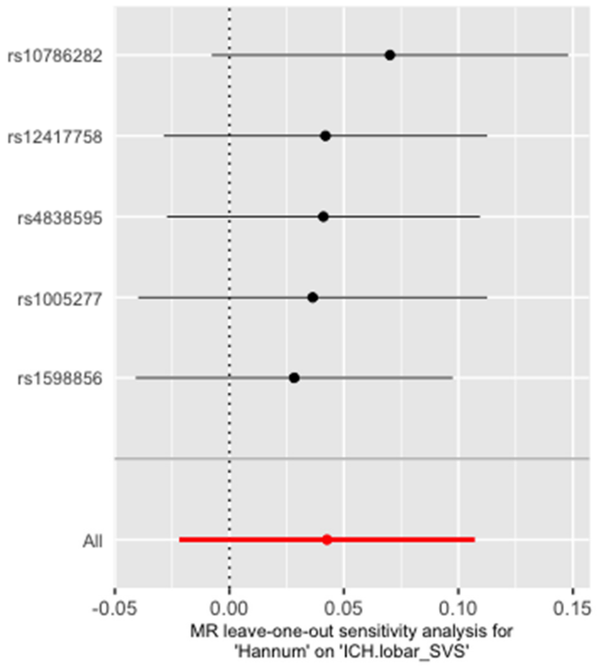


Supplement 2 Figure 40. The single SNP analysis and leave-one-out analysis for HannumAge on non-lobar ICH or SVS

(a) Forest plot of single SNP MR

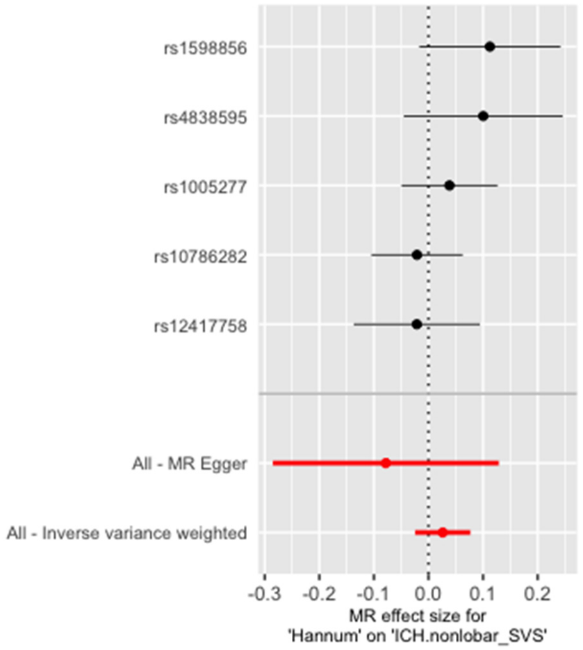


(b) Leave-one-out analysis

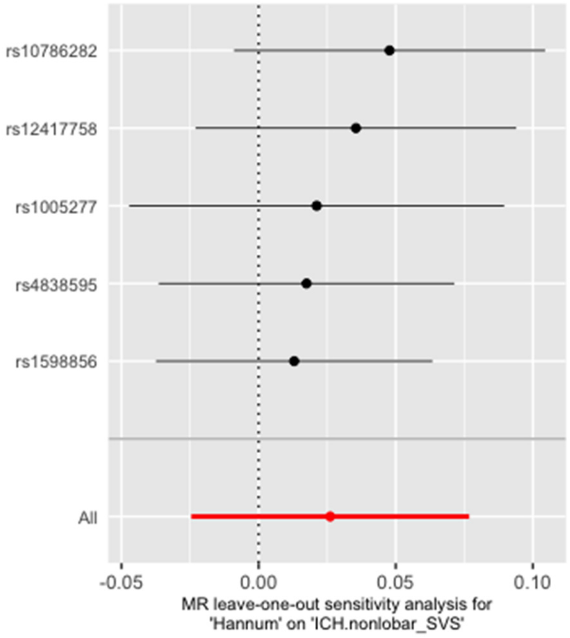


Supplement 2 Figure 41. The single SNP analysis and leave-one-out analysis for Intrinsic HorvathAge acceleration on WMH volume

(a) Forest plot of single SNP MR

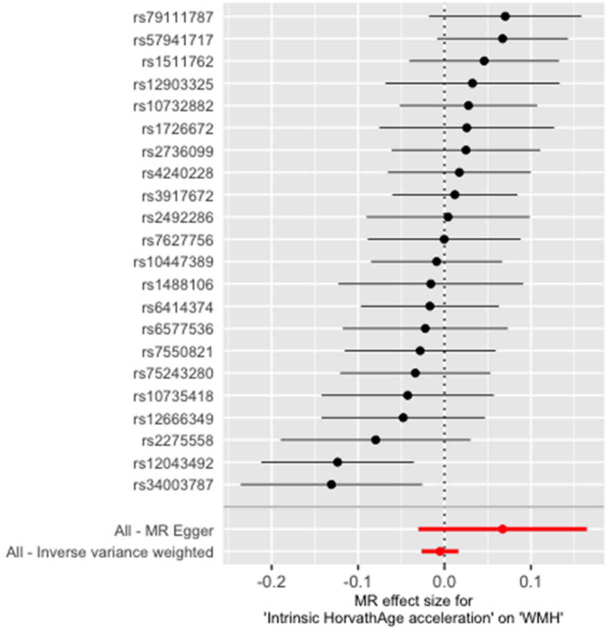


(b) Leave-one-out analysis

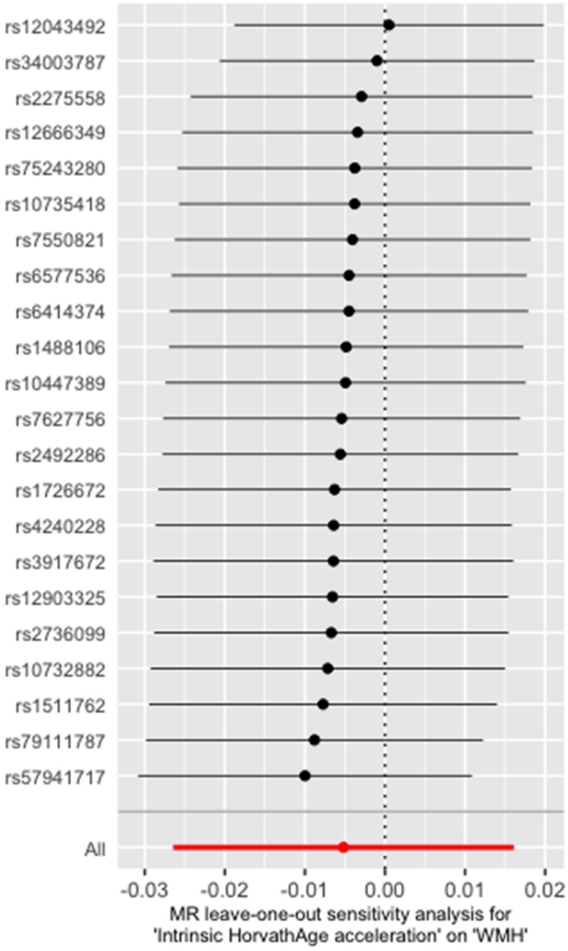


Supplement 2 Figure 42. The single SNP analysis and leave-one-out analysis for Intrinsic HorvathAge acceleration on FA

(a) Forest plot of single SNP MR

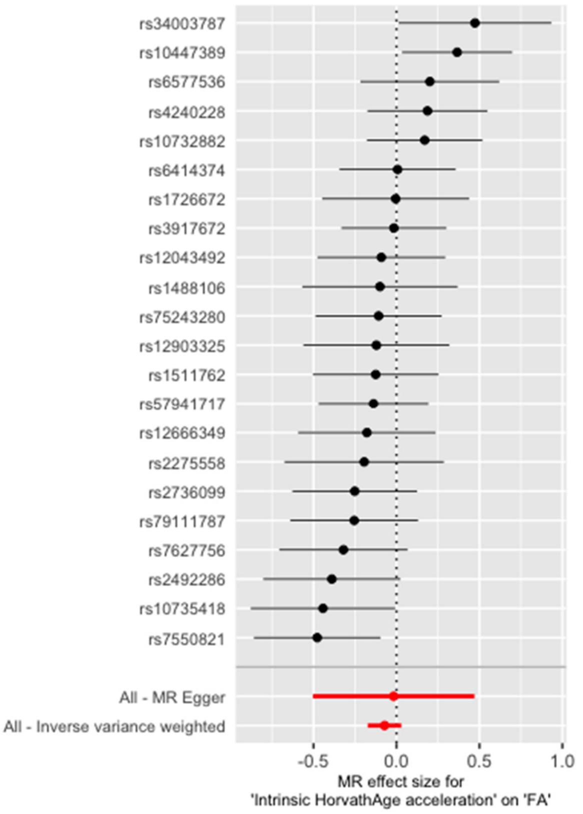


(b) Leave-one-out analysis

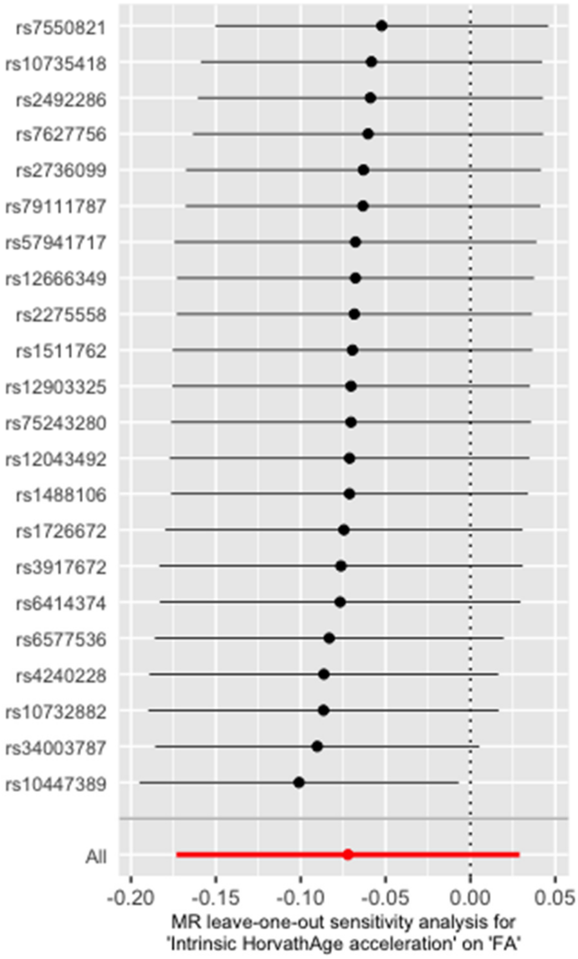

Supplement 2 Figure 43. The single SNP analysis and leave-one-out analysis for Intrinsic HorvathAge acceleration on MD

(a) Forest plot of single SNP MR

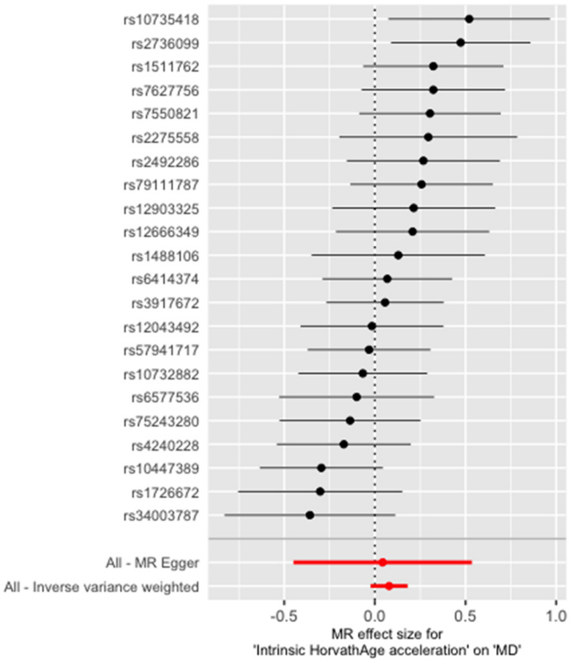


(b) Leave-one-out analysis

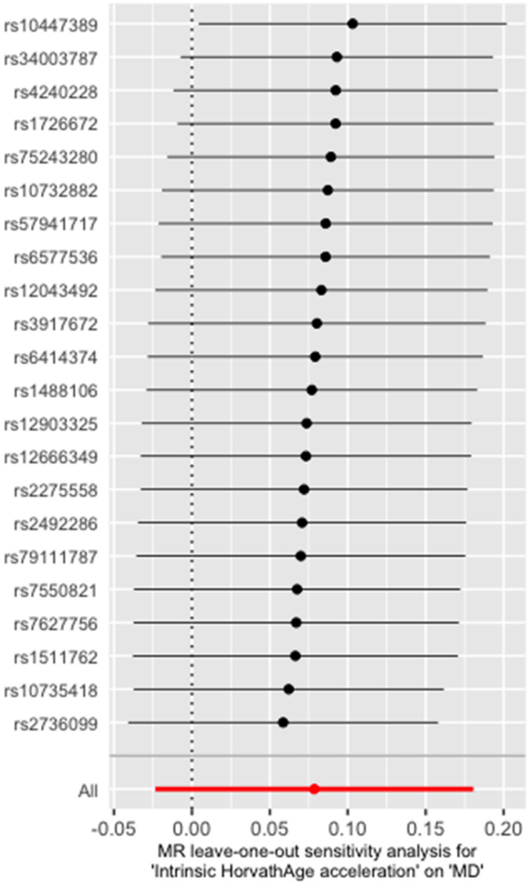

Supplement 2 Figure 44. The single SNP analysis and leave-one-out analysis for Intrinsic HorvathAge acceleration on lacunar stroke

(a) Forest plot of single SNP MR

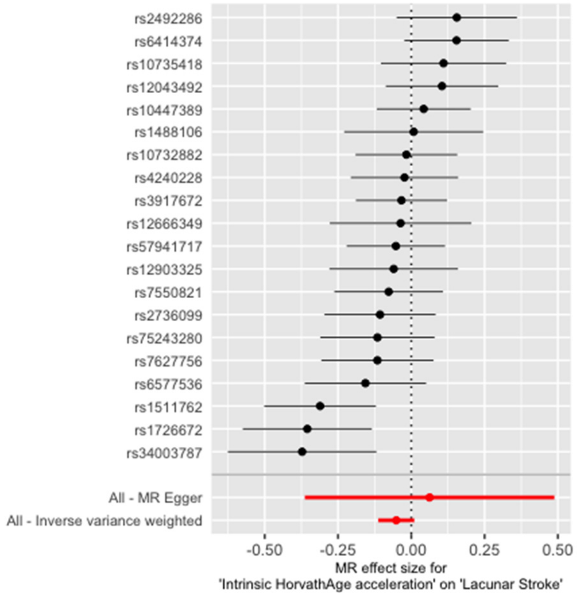


(b) Leave-one-out analysis

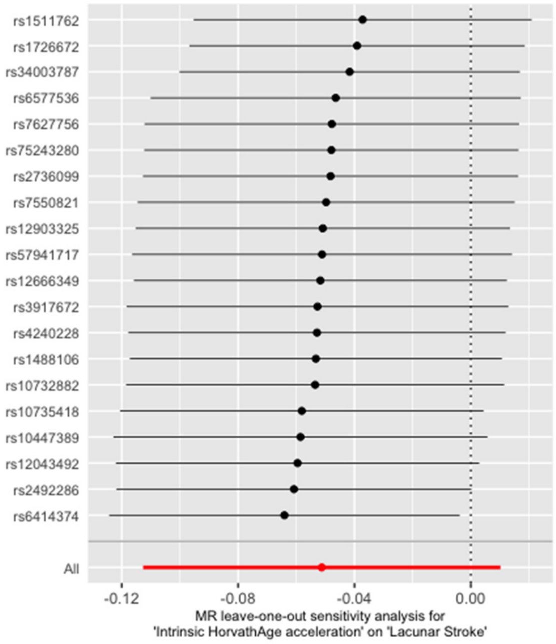


Supplement 2 Figure 45. The single SNP analysis and leave-one-out analysis for Intrinsic HorvathAge acceleration on all location BMB

(a) Forest plot of single SNP MR

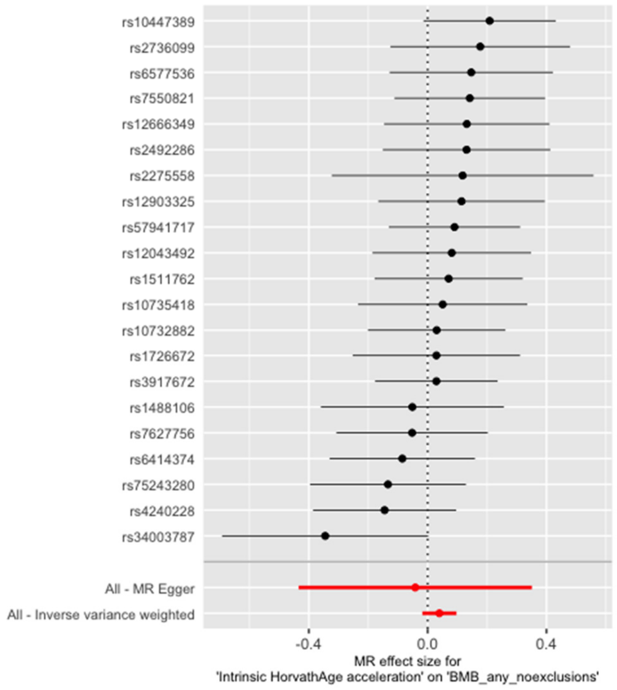


(b) Leave-one-out analysis

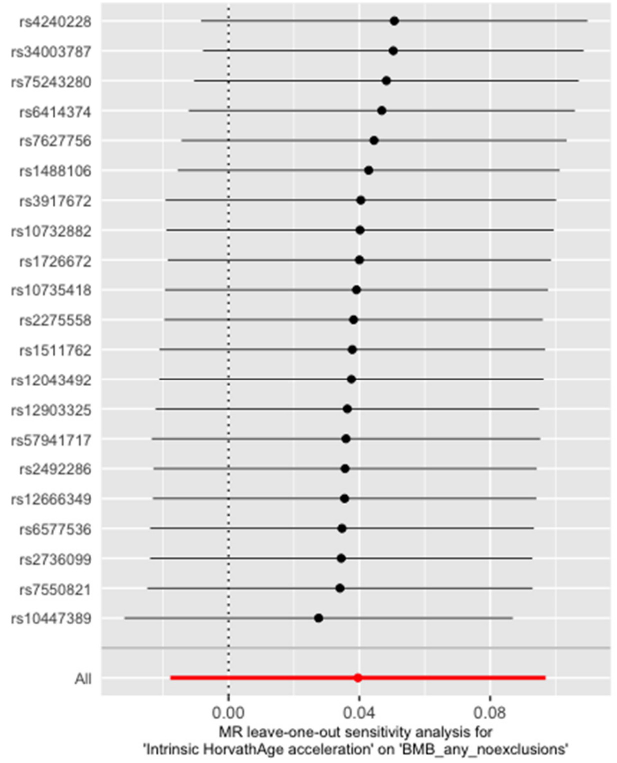


Supplement 2 Figure 46. The single SNP analysis and leave-one-out analysis for Intrinsic HorvathAge acceleration on lobar BMB

(a) Forest plot of single SNP MR

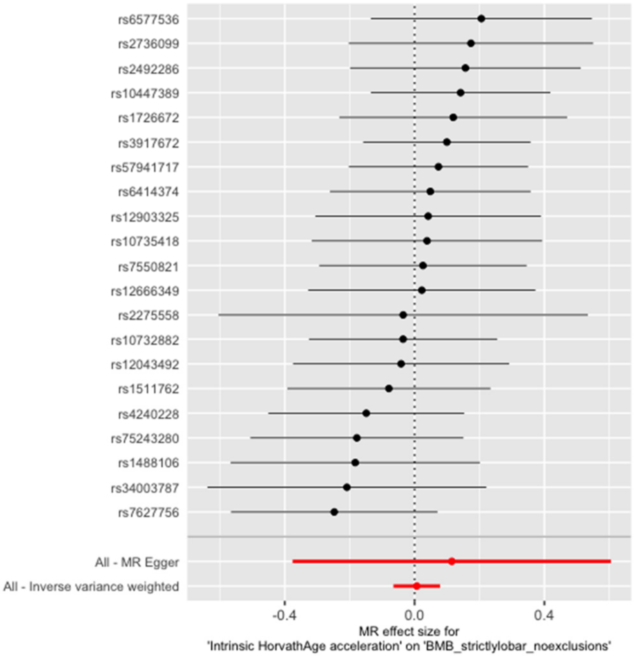


(b) Leave-one-out analysis

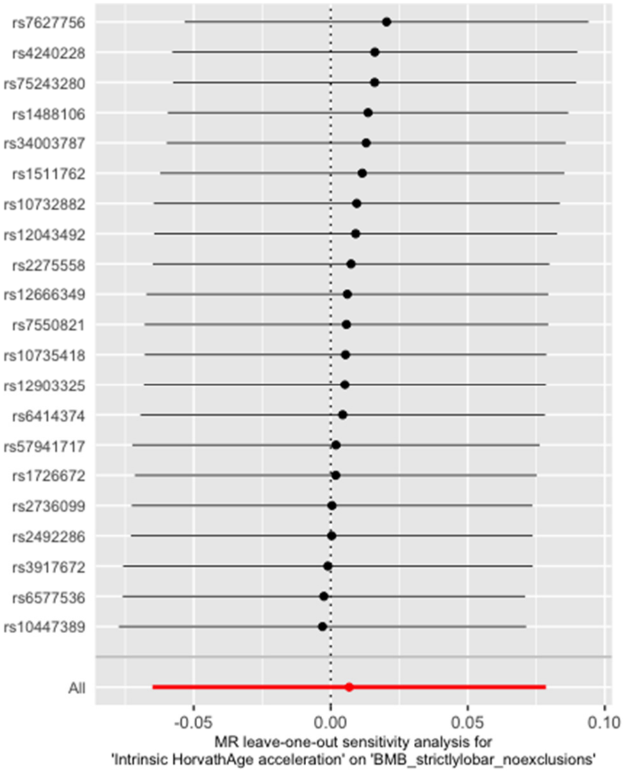


Supplement 2 Figure 47. The single SNP analysis and leave-one-out analysis for Intrinsic HorvathAge acceleration on mixed or deep BMB

(a) Forest plot of single SNP MR

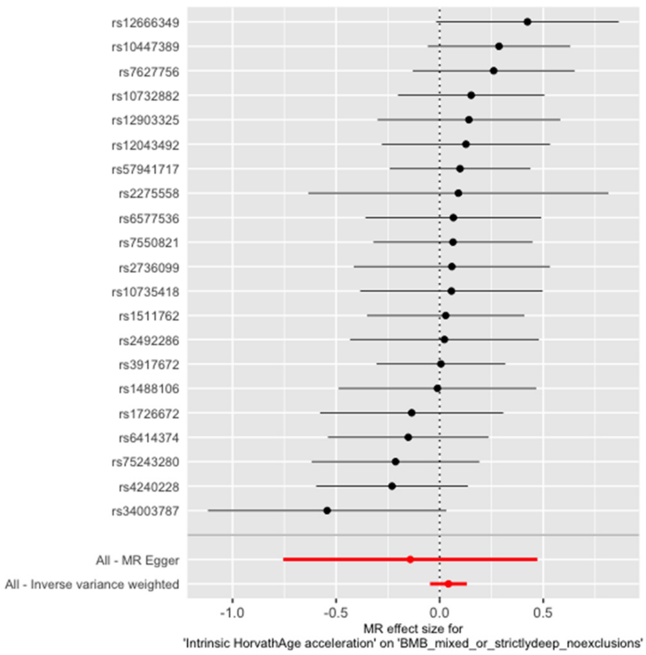


(b) Leave-one-out analysis

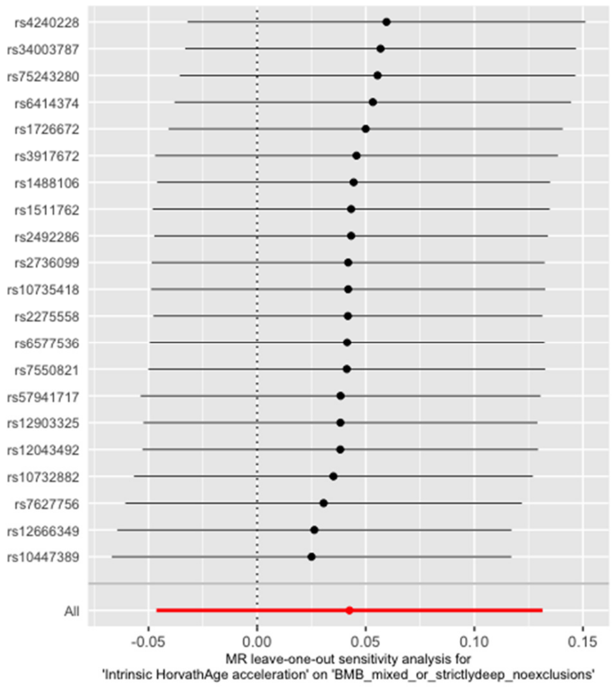


Supplement 2 Figure 48. The single SNP analysis and leave-one-out analysis for Intrinsic HorvathAge acceleration on all location ICH or SVS

(a) Forest plot of single SNP MR

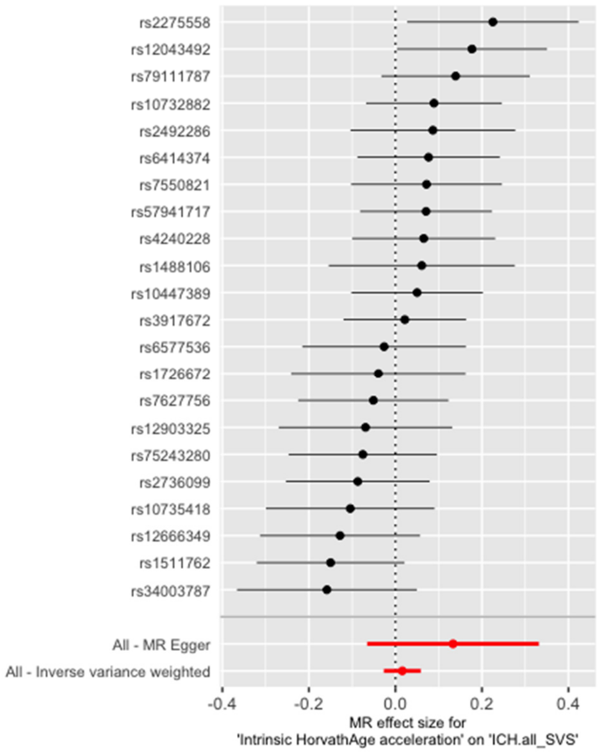


(b) Leave-one-out analysis

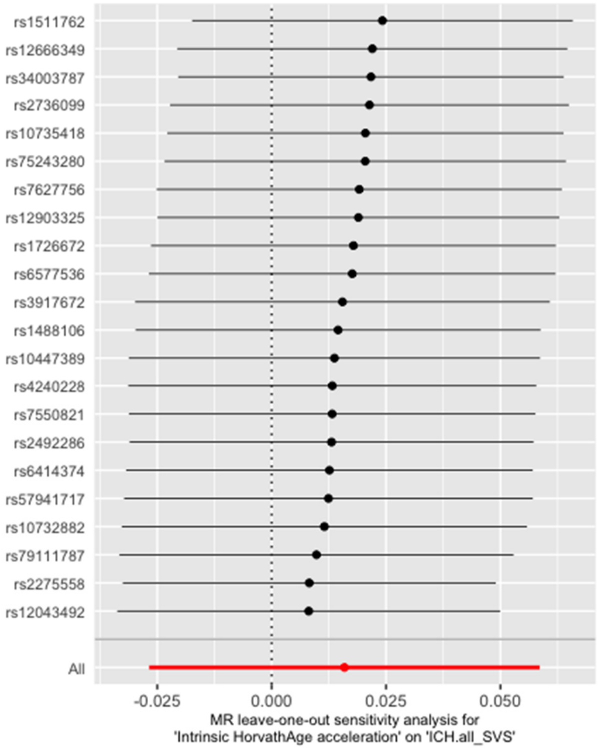


Supplement 2 Figure 49. The single SNP analysis and leave-one-out analysis for Intrinsic HorvathAge acceleration on lobar ICH or SVS

(a) Forest plot of single SNP MR

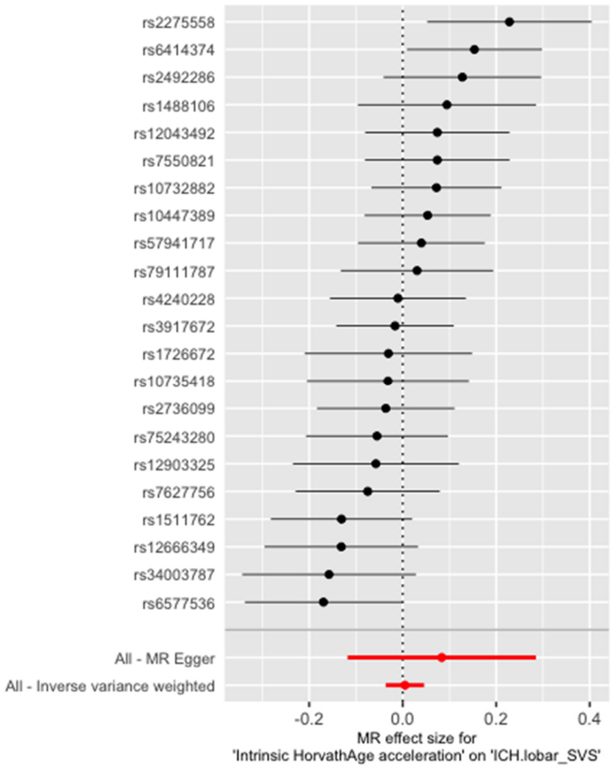


(b) Leave-one-out analysis

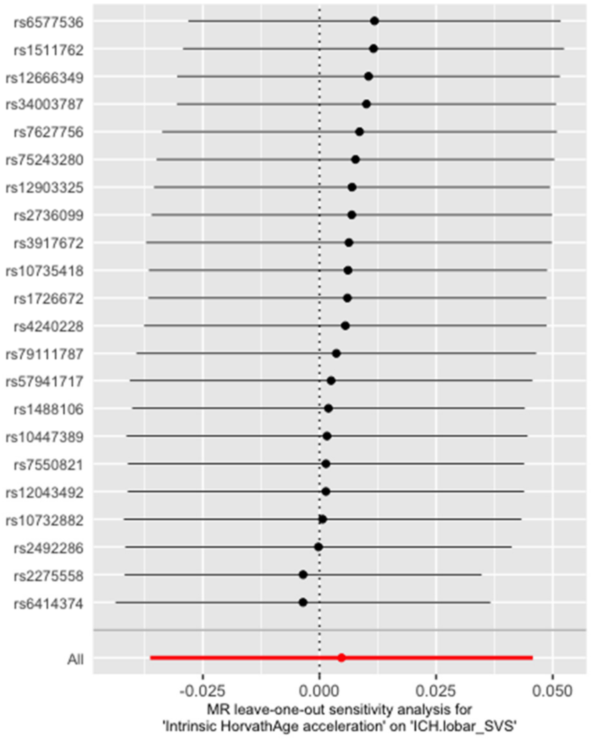


Supplement 2 Figure 50. The single SNP analysis and leave-one-out analysis for Intrinsic HorvathAge acceleration on non-lobar ICH or SVS

(a) Forest plot of single SNP MR

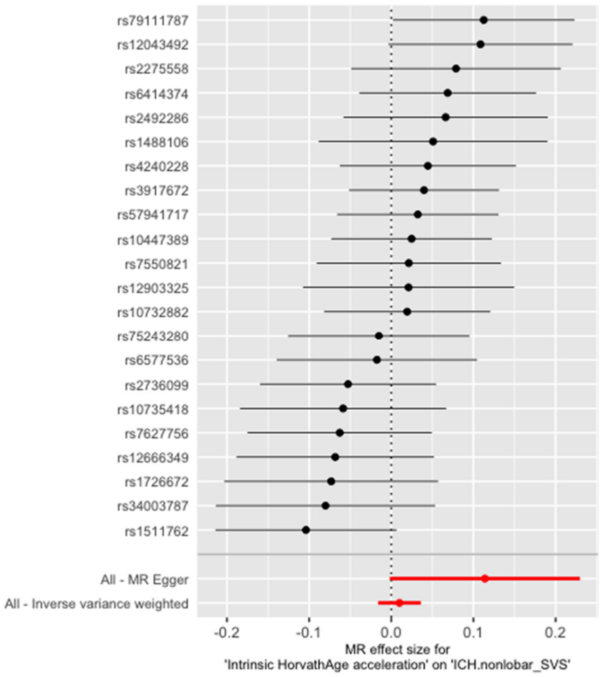


(b) Leave-one-out analysis

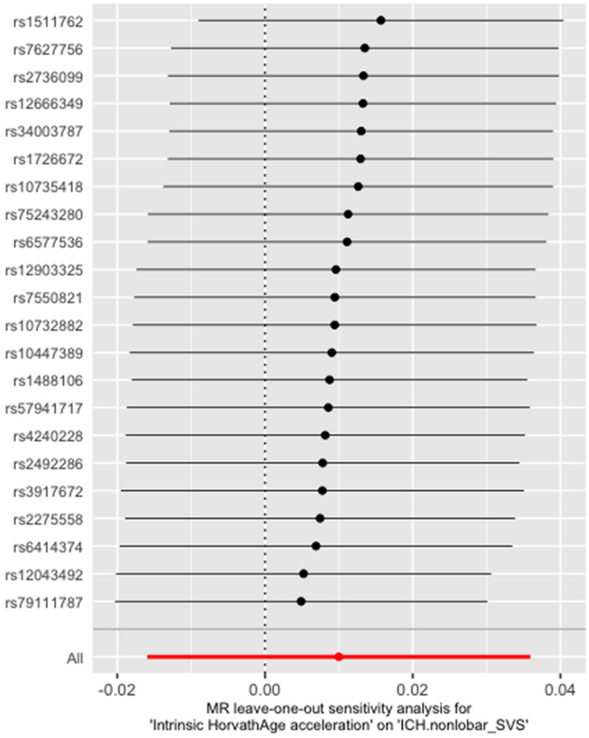


## Supplement 3. Results of other four MR methods for causal effect of exposures on outcomes

Supplement 3 Table 1. Results of other four MR methods for causal effect of LTL on CSVD

| TL on CSVD | No.of SNPs | Method | OR | [95%CI] | P |
|---|---|---|---|---|---|

| | | | | | |
|---|---|---|---|---|---|
| WMH volume | 119 | MR Egger | 1.07 | [0.93, 1.24] | 0.32 |
| | | Weighted median | 1.00 | [0.88, 1.15] | 0.94 |
| | | Simple mode | 1.07 | [0.80, 1.44] | 0.64 |
| | | Weighted mode | 1.01 | [0.84, 1.20] | 0.95 |
| FA | 119 | MR Egger | 0.41 | [0.21, 0.81] | 0.01 |
| | | Weighted median | 0.76 | [0.43, 1.36] | 0.36 |
| | | Simple mode | 0.77 | [0.26, 2.24] | 0.63 |
| | | Weighted mode | 0.70 | [0.37, 1.34] | 0.29 |
| MD | 118 | MR Egger | 1.57 | [0.79, 3.11] | 0.20 |
| | | Weighted median | 1.64 | [0.91, 2.95] | 0.10 |
| | | Simple mode | 1.61 | [0.51, 5.14] | 0.42 |
| | | Weighted mode | 1.61 | [0.82, 3.16] | 0.17 |
| lacunar stroke | 98 | MR Egger | 0.76 | [0.50, 1.14] | 0.19 |
| | | Weighted median | 0.99 | [0.74, 1.33] | 0.96 |
| | | Simple mode | 0.80 | [0.46, 1.37] | 0.41 |
| | | Weighted mode | 0.98 | [0.69, 1.39] | 0.92 |
| all location BMB | 94 | MR Egger | 0.82 | [0.48, 1.40] | 0.46 |
| | | Weighted median | 1.01 | [0.66, 1.55] | 0.97 |
| | | Simple mode | 1.06 | [0.48, 2.32] | 0.89 |
| | | Weighted mode | 1.03 | [0.64, 1.65] | 0.91 |
| lobar BMB | 94 | MR Egger | 0.97 | [0.49, 1.92] | 0.94 |
| | | Weighted median | 0.91 | [0.54, 1.53] | 0.72 |
| | | Simple mode | 1.04 | [0.38, 2.79] | 0.95 |
| | | Weighted mode | 0.88 | [0.50, 1.56] | 0.66 |
| mixed or deep BMB | 89 | MR Egger | 0.69 | [0.27, 1.72] | 0.42 |
| | | Weighted median | 1.36 | [0.67, 2.77] | 0.40 |
| | | Simple mode | 1.89 | [0.52, 6.87] | 0.34 |
| | | Weighted mode | 1.53 | [0.68, 3.44] | 0.31 |
| all location ICH or SVS | 98 | MR Egger | 0.92 | [0.65, 1.31] | 0.66 |
| | | Weighted median | 1.09 | [0.83, 1.44] | 0.53 |
| | | Simple mode | 1.12 | [0.62, 2.01] | 0.70 |
| | | Weighted mode | 1.14 | [0.76, 1.72] | 0.52 |
| lobar ICH or SVS | 101 | MR Egger | 0.96 | [0.73, 1.27] | 0.77 |
| | | Weighted median | 1.04 | [0.81, 1.35] | 0.74 |
| | | Simple mode | 1.42 | [0.84, 2.39] | 0.20 |
| | | Weighted mode | 1.11 | [0.81, 1.52] | 0.52 |
| non-lobar ICH or SVS | 97 | MR Egger | 0.95 | [0.76, 1.18] | 0.62 |
| | | Weighted median | 1.04 | [0.85, 1.26] | 0.72 |
| | | Simple mode | 1.19 | [0.79, 1.79] | 0.42 |

| | | | | | |
|---|---|---|---|---|---|
| | | Weighted mode | 1.07 | [0.81, 1.40] | 0.63 |

Supplement 3 Table 2. Results of other four MR methods for causal effect of GrimAge on CSVD

| GrimAge on CSVD | No.of SNPs | Method | OR | [95%CI] | P |
|---|---|---|---|---|---|
| WMH volume | 4 | MR Egger | 4.41 | [1.84, 10.57] | 0.08 |
| | | Weighted median | 1.00 | [0.92, 1.08] | 0.98 |
| | | Simple mode | 0.98 | [0.85, 1.13] | 0.81 |
| | | Weighted mode | 1.00 | [0.87, 1.15] | 0.99 |
| FA | 4 | MR Egger | 0.01 | [0.00, 1.14] | 0.20 |
| | | Weighted median | 1.12 | [0.83, 1.53] | 0.45 |
| | | Simple mode | 1.21 | [0.69, 2.11] | 0.55 |
| | | Weighted mode | 1.20 | [0.68, 2.11] | 0.57 |
| MD | 4 | MR Egger | 9.12 | [0.18, 455.84] | 0.38 |
| | | Weighted median | 1.01 | [0.76, 1.36] | 0.93 |
| | | Simple mode | 1.12 | [0.72, 1.73] | 0.65 |
| | | Weighted mode | 1.12 | [0.75, 1.66] | 0.63 |
| lacunar stroke | 4 | MR Egger | 0.42 | [0.07, 2.64] | 0.45 |
| | | Weighted median | 0.97 | [0.85, 1.10] | 0.62 |
| | | Simple mode | 0.98 | [0.82, 1.18] | 0.84 |
| | | Weighted mode | 0.98 | [0.80, 1.19] | 0.84 |
| all location BMB | 3 | MR Egger | 0.24 | [0.00, 142.24] | 0.74 |
| | | Weighted median | 1.06 | [0.82, 1.37] | 0.65 |
| | | Simple mode | 1.02 | [0.70, 1.48] | 0.94 |
| | | Weighted mode | 1.05 | [0.75, 1.45] | 0.81 |
| lobar BMB | 3 | MR Egger | 0.04 | [8.86E-6, 198.45] | 0.60 |
| | | Weighted median | 0.99 | [7.23E-1, 1.35] | 0.95 |
| | | Simple mode | 0.89 | [6.02E-1, 1.31] | 0.61 |
| | | Weighted mode | 0.92 | [6.50E-1, 1.31] | 0.70 |
| mixed or deep BMB | 3 | MR Egger | 2.94 | [0.01, 679.25] | 0.76 |
| | | Weighted median | 1.14 | [0.82, 1.60] | 0.42 |
| | | Simple mode | 1.17 | [0.79, 1.74] | 0.52 |
| | | Weighted mode | 1.18 | [0.79, 1.75] | 0.50 |
| all location ICH or SVS | 3 | MR Egger | 0.07 | [0.01, 0.46] | 0.22 |
| | | Weighted median | 0.94 | [0.78, 1.13] | 0.50 |
| | | Simple mode | 0.95 | [0.71, 1.28] | 0.78 |
| | | Weighted mode | 0.89 | [0.67, 1.20] | 0.53 |
| lobar ICH or SVS | 3 | MR Egger | 0.06 | [0.01, 0.31] | 0.19 |

| | | | | | |
|---|---|---|---|---|---|
| | | Weighted median | 1.02 | [0.87, 1.20] | 0.79 |
| | | Simple mode | 1.12 | [0.88, 1.41] | 0.46 |
| | | Weighted mode | 1.11 | [0.85, 1.45] | 0.52 |
| non-lobar ICH or SVS | 3 | MR Egger | 0.36 | [0.11, 1.20] | 0.34 |
| | | Weighted median | 0.96 | [0.87, 1.06] | 0.44 |
| | | Simple mode | 0.98 | [0.85, 1.13] | 0.79 |
| | | Weighted mode | 0.94 | [0.82, 1.08] | 0.48 |

Supplement 3 Table 3. Results of other four MR methods for causal effect of PhenoAge on CSVD.

| PhenoAge on CSVD | No.of SNPs | Method | OR | [95%CI] | P |
|---|---|---|---|---|---|
| WMH volume | 9 | MR Egger | 1.02 | [0.93, 1.11] | 0.69 |
| | | Weighted median | 1.02 | [0.99, 1.06] | 0.15 |
| | | Simple mode | 1.03 | [0.98, 1.08] | 0.31 |
| | | Weighted mode | 1.03 | [0.98, 1.08] | 0.25 |
| FA | 9 | MR Egger | 0.98 | [0.67, 1.44] | 0.92 |
| | | Weighted median | 1.01 | [0.89, 1.16] | 0.84 |
| | | Simple mode | 1.06 | [0.85, 1.31] | 0.64 |
| | | Weighted mode | 1.02 | [0.83, 1.25] | 0.86 |
| MD | 9 | MR Egger | 1.05 | [0.71, 1.55] | 0.82 |
| | | Weighted median | 0.88 | [0.77, 1.01] | 0.08 |
| | | Simple mode | 0.87 | [0.68, 1.09] | 0.26 |
| | | Weighted mode | 0.87 | [0.71, 1.07] | 0.23 |
| lacunar stroke | 7 | MR Egger | 0.91 | [0.74, 1.05] | 0.39 |
| | | Weighted median | 0.97 | [0.90, 1.05] | 0.48 |
| | | Simple mode | 0.96 | [0.87, 1.07] | 0.52 |
| | | Weighted mode | 0.96 | [0.87, 1.07] | 0.48 |
| all location BMB | 9 | MR Egger | 1.04 | [0.77, 1.40] | 0.80 |
| | | Weighted median | 0.95 | [0.86, 1.04] | 0.26 |
| | | Simple mode | 0.93 | [0.81, 1.06] | 0.29 |
| | | Weighted mode | 0.93 | [0.82, 1.05] | 0.27 |
| lobar BMB | 9 | MR Egger | 1.09 | [0.78, 1.52] | 0.64 |
| | | Weighted median | 0.98 | [0.87, 1.10] | 0.70 |
| | | Simple mode | 0.99 | [0.83, 1.19] | 0.94 |
| | | Weighted mode | 0.93 | [0.79, 1.10] | 0.43 |
| mixed or deep BMB | 9 | MR Egger | 0.93 | [0.59, 1.44] | 0.75 |
| | | Weighted median | 0.90 | [0.78, 1.03] | 0.13 |
| | | Simple mode | 0.85 | [0.69, 1.06] | 0.18 |
| | | Weighted mode | 0.87 | [0.72, 1.06] | 0.20 |

| | | | | | |
|---|---|---|---|---|---|
| all location ICH or SVS | 9 | MR Egger | 0.98 | [0.82, 1.17] | 0.80 |
| | | Weighted median | 0.98 | [0.92, 1.03] | 0.40 |
| | | Simple mode | 0.96 | [0.87, 1.06] | 0.49 |
| | | Weighted mode | 0.96 | [0.88, 1.05] | 0.38 |
| lobar ICH or SVS | 9 | MR Egger | 1.04 | [0.89, 1.22] | 0.62 |
| | | Weighted median | 0.98 | [0.93, 1.04] | 0.51 |
| | | Simple mode | 0.98 | [0.90, 1.06] | 0.58 |
| | | Weighted mode | 0.98 | [0.90, 1.06] | 0.57 |
| non-lobar ICH or SVS | 9 | MR Egger | 0.93 | [0.81, 1.07] | 0.36 |
| | | Weighted median | 0.97 | [0.93, 1.01] | 0.19 |
| | | Simple mode | 0.97 | [0.91, 1.03] | 0.38 |
| | | Weighted mode | 0.97 | [0.91, 1.02] | 0.29 |

Supplement 3 Table 4. Results of other four MR methods for causal effect of HannumAge on CSVD

| HannumAge on CSVD | No.of SNPs | Method | OR | [95%CI] | P |
|---|---|---|---|---|---|
| WMH volume | 5 | MR Egger | 0.90 | [0.78, 1.05] | 0.28 |
| | | Weighted median | 0.97 | [0.92, 1.01] | 0.13 |
| | | Simple mode | 0.96 | [0.90, 1.02] | 0.27 |
| | | Weighted mode | 0.96 | [0.91, 1.01] | 0.22 |
| FA | 5 | MR Egger | 0.84 | [0.44, 1.62] | 0.64 |
| | | Weighted median | 0.96 | [0.79, 1.17] | 0.71 |
| | | Simple mode | 0.98 | [0.75, 1.28] | 0.90 |
| | | Weighted mode | 0.94 | [0.73, 1.22] | 0.68 |
| MD | 5 | MR Egger | 0.85 | [0.34, 2.13] | 0.75 |
| | | Weighted median | 1.04 | [0.85, 1.28] | 0.70 |
| | | Simple mode | 0.98 | [0.76, 1.27] | 0.90 |
| | | Weighted mode | 0.99 | [0.78, 1.25] | 0.94 |
| lacunar stroke | 5 | MR Egger | 0.85 | [0.62, 1.17] | 0.40 |
| | | Weighted median | 0.97 | [0.88, 1.08] | 0.60 |
| | | Simple mode | 0.96 | [0.83, 1.11] | 0.58 |
| | | Weighted mode | 0.94 | [0.81, 1.09] | 0.48 |
| all location BMB | 5 | MR Egger | 0.90 | [0.53, 1.52] | 0.72 |
| | | Weighted median | 1.07 | [0.94, 1.22] | 0.32 |
| | | Simple mode | 1.09 | [0.88, 1.34] | 0.47 |
| | | Weighted mode | 1.08 | [0.89, 1.32] | 0.46 |
| lobar BMB | 5 | MR Egger | 0.88 | [0.51, 1.52] | 0.68 |
| | | Weighted median | 1.07 | [0.91, 1.25] | 0.44 |
| | | Simple mode | 1.10 | [0.89, 1.35] | 0.42 |

| | | | | | |
|---|---|---|---|---|---|
| | | Weighted mode | 1.07 | [0.88, 1.30] | 0.53 |
| mixed or deep BMB | 5 | MR Egger | 1.73 | [0.88, 3.38] | 0.21 |
| | | Weighted median | 0.89 | [0.71, 1.11] | 0.31 |
| | | Simple mode | 0.84 | [0.60, 1.16] | 0.35 |
| | | Weighted mode | 0.86 | [0.58, 1.26] | 0.48 |
| all location ICH or SVS | 5 | MR Egger | 0.90 | [0.67, 1.22] | 0.55 |
| | | Weighted median | 1.07 | [0.98, 1.18] | 0.12 |
| | | Simple mode | 1.07 | [0.95, 1.22] | 0.34 |
| | | Weighted mode | 1.07 | [0.96, 1.21] | 0.29 |
| lobar ICH or SVS | 5 | MR Egger | 0.89 | [0.68, 1.16] | 0.45 |
| | | Weighted median | 1.05 | [0.97, 1.14] | 0.19 |
| | | Simple mode | 1.05 | [0.93, 1.19] | 0.44 |
| | | Weighted mode | 1.06 | [0.95, 1.17] | 0.36 |
| non-lobar ICH or SVS | 5 | MR Egger | 0.92 | [0.75, 1.14] | 0.51 |
| | | Weighted median | 1.01 | [0.96, 1.07] | 0.66 |
| | | Simple mode | 1.00 | [0.91, 1.09] | 0.95 |
| | | Weighted mode | 1.00 | [0.92, 1.08] | 0.95 |

Supplement 3 Table 5. Results of other four MR methods for causal effect of Intrinsic HorvathAge acceleration on CSVD

| Intrinsic HorvathAge acceleration on CSVD | No.of SNPs | Method | OR | [95%CI] | P |
|---|---|---|---|---|---|
| WMH volume | 22 | MR Egger | 1.07 | [0.97, 1.18] | 0.19 |
| | | Weighted median | 1.00 | [0.97, 1.03] | 0.92 |
| | | Simple mode | 1.00 | [0.95, 1.05] | 0.94 |
| | | Weighted mode | 1.00 | [0.96, 1.05] | 0.85 |
| FA | 22 | MR Egger | 0.98 | [0.60, 1.60] | 0.95 |
| | | Weighted median | 0.90 | [0.79, 1.02] | 0.09 |
| | | Simple mode | 0.88 | [0.69, 1.12] | 0.32 |
| | | Weighted mode | 0.89 | [0.71, 1.12] | 0.34 |
| MD | 22 | MR Egger | 1.04 | [0.64, 1.71] | 0.87 |
| | | Weighted median | 1.06 | [0.93, 1.21] | 0.35 |
| | | Simple mode | 1.28 | [0.96, 1.69] | 0.10 |
| | | Weighted mode | 1.00 | [0.77, 1.30] | 0.99 |
| lacunar stroke | 20 | MR Egger | 1.06 | [0.70, 1.63] | 0.78 |
| | | Weighted median | 0.97 | [0.91, 1.03] | 0.28 |
| | | Simple mode | 0.95 | [0.85, 1.06] | 0.38 |

| | | | | | |
|---|---|---|---|---|---|
| | | Weighted mode | 0.95 | [0.86, 1.06] | 0.37 |
| all location BMB | 21 | MR Egger | 0.96 | [0.65, 1.42] | 0.84 |
| | | Weighted median | 1.06 | [0.98, 1.15] | 0.12 |
| | | Simple mode | 1.11 | [0.97, 1.28] | 0.16 |
| | | Weighted mode | 1.09 | [0.94, 1.26] | 0.26 |
| lobar BMB | 21 | MR Egger | 1.12 | [0.69, 1.83] | 0.65 |
| | | Weighted median | 1.04 | [0.94, 1.14] | 0.46 |
| | | Simple mode | 1.05 | [0.87, 1.26] | 0.61 |
| | | Weighted mode | 1.06 | [0.90, 1.26] | 0.48 |
| mixed or deep BMB | 21 | MR Egger | 0.87 | [0.47, 1.60] | 0.66 |
| | | Weighted median | 1.06 | [0.94, 1.20] | 0.33 |
| | | Simple mode | 1.07 | [0.86, 1.33] | 0.58 |
| | | Weighted mode | 1.06 | [0.85, 1.33] | 0.60 |
| all location ICH or SVS | 22 | MR Egger | 1.14 | [0.94, 1.39] | 0.20 |
| | | Weighted median | 1.05 | [0.99, 1.10] | 0.09 |
| | | Simple mode | 1.07 | [0.96, 1.19] | 0.25 |
| | | Weighted mode | 1.07 | [0.96, 1.18] | 0.23 |
| lobar ICH or SVS | 22 | MR Egger | 1.09 | [0.89, 1.33] | 0.43 |
| | | Weighted median | 0.99 | [0.94, 1.04] | 0.67 |
| | | Simple mode | 0.99 | [0.89, 1.09] | 0.80 |
| | | Weighted mode | 1.00 | [0.92, 1.09] | 0.95 |
| non-lobar ICH or SVS | 22 | MR Egger | 1.12 | [1.00, 1.26] | 0.07 |
| | | Weighted median | 1.02 | [0.99, 1.06] | 0.21 |
| | | Simple mode | 1.04 | [0.97, 1.11] | 0.31 |
| | | Weighted mode | 1.04 | [0.97, 1.10] | 0.27 |

**Supplement 4. Results of sensitivity analysis**

Supplement 4 Table 1. Results of sensitivity analysis of LTL on CSVD

| LTL on CSVD | Heterogeneity test (Cochran's Q) | | Pleiotropy test | MR PRESSO | | F |
|---|---|---|---|---|---|---|
| | P (MR Egger) | P(IVW) | P (MR Egger) | P(Global test) | Outlier | |
| WMH volume | 0.40 | 0.41 | 0.58 | 0.18 | NA | 36.87 |
| FA | 0.06 | 0.03 | 0.02 | 0.09 | NA | 36.87 |
| MD | 0.22 | 0.24 | 0.58 | 0.28 | NA | 35.62 |
| lacunar stroke | 0.003 | 0.002 | 0.08 | 0.003 | 3 | 39.06 |
| all location | 0.25 | 0.22 | 0.16 | 0.18 | NA | 44.21 |

| | | | | | | |
|---|---|---|---|---|---|---|
| BMB | | | | | | |
| lobar BMB | 0.26 | 0.29 | 0.83 | 0.27 | NA | 44.21 |
| mixed or deep BMB | 0.07 | 0.048 | 0.09 | 0.04 | NA | 46.31 |
| all location ICH or SVS | 0.01 | 0.01 | 0.89 | 0.008 | NA | 38.06 |
| lobar ICH or SVS | 0.19 | 0.21 | 0.99 | 0.17 | NA | 37.23 |
| non-lobar ICH or SVS | 0.03 | 0.04 | 0.68 | 0.02 | NA | 38.44 |

Supplement 4 Table 2. Results of sensitivity analysis of GrimAge on CSVD

| GrimAge on CSVD | Heterogeneity test (Cochran's Q) | | Pleiotropy test | MR PRESSO | | F |
|---|---|---|---|---|---|---|
| | P (MR Egger) | P(IVW) | P (MR Egger) | P(Global test) | Outlier | |
| WMH volume | 0.59 | 0.01 | 0.08 | 0.04 | 2 | 17.58 |
| FA | 0.27 | 0.05 | 0.19 | 0.11 | NA | 17.58 |
| MD | 0.45 | 0.41 | 0.38 | 0.48 | NA | 17.58 |
| lacunar stroke | 0.86 | 0.79 | 0.48 | 0.78 | NA | 17.58 |
| all location BMB | 0.02 | 0.03 | 0.73 | - | - | 17.34 |
| lobar BMB | 0.01 | 0.01 | 0.58 | - | - | 17.34 |
| mixed or deep BMB | 0.19 | 0.38 | 0.77 | - | - | 17.34 |
| all location ICH or SVS | 0.71 | 0.02 | 0.22 | - | - | 17.34 |
| lobar ICH or SVS | 0.81 | 0.003 | 0.87 | - | - | 17.34 |
| non-lobar ICH or SVS | 0.87 | 0.27 | 0.35 | - | - | 17.34 |

- means MR PRESSO is not suitable to carry out

Supplement 4 Table 3. Results of sensitivity analysis of PhenoAge on CSVD

| PhenoAge on CSVD | Heterogeneity test (Cochran's Q) | | Pleiotropy test | MR PRESSO | | F |
|---|---|---|---|---|---|---|
| | P (MR Egger) | P(IVW) | P (MR Egger) | P(Global test) | Outlier | |
| WMH volume | 0.45 | 0.55 | 0.86 | 0.62 | NA | 14.17 |
| FA | 0.89 | 0.93 | 0.81 | 0.93 | NA | 14.17 |

| | | | | | | |
|---|---|---|---|---|---|---|
| MD | 0.81 | 0.85 | 0.57 | 0.87 | NA | 14.17 |
| lacunar stroke | 0.36 | 0.37 | 0.39 | 0.46 | NA | 12.92 |
| all location BMB | 0.24 | 0.31 | 0.63 | 0.45 | NA | 14.17 |
| lobar BMB | 0.51 | 0.58 | 0.57 | 0.65 | NA | 14.17 |
| mixed or deep BMB | 0.88 | 0.93 | 0.93 | 0.98 | NA | 14.17 |
| all location ICH or SVS | 0.64 | 0.73 | 0.93 | 0.67 | NA | 14.17 |
| lobar ICH or SVS | 0.81 | 0.85 | 0.60 | 0.89 | NA | 14.17 |
| non-lobar ICH or SVS | 0.18 | 0.20 | 0.45 | 0.17 | NA | 14.17 |

Supplement 4 Table 4. Results of sensitivity analysis of HannumAge on CSVD

| HannumAge on CSVD | Heterogeneity test (Cochran's Q) | | Pleiotropy test | MR PRESSO | | F |
|---|---|---|---|---|---|---|
| | P (MR Egger) | P(IVW) | P (MR Egger) | P(Global test) | Outlier | |
| WMH volume | 0.67 | 0.63 | 0.38 | 0.54 | NA | 23.62 |
| FA | 0.46 | 0.62 | 0.82 | 0.50 | NA | 23.62 |
| MD | 0.13 | 0.19 | 0.65 | 0.05 | NA | 23.62 |
| lacunar stroke | 0.60 | 0.60 | 0.41 | 0.59 | NA | 23.62 |
| all location BMB | 0.22 | 0.33 | 0.73 | 0.53 | NA | 23.62 |
| lobar BMB | 0.66 | 0.72 | 0.53 | 0.84 | NA | 23.62 |
| mixed or deep BMB | 0.42 | 0.21 | 0.18 | 0.26 | NA | 23.62 |
| all location ICH or SVS | 0.82 | 0.71 | 0.35 | 0.32 | NA | 23.62 |
| lobar ICH or SVS | 0.87 | 0.69 | 0.31 | 0.62 | NA | 23.62 |
| non-lobar ICH or SVS | 0.33 | 0.32 | 0.38 | 0.33 | NA | 23.62 |

Supplement 4 Table 5. Results of sensitivity analysis of Intrinsic HorvathAge acceleration on CSVD

| Intrinsic HorvathAge acceleration on CSVD | Heterogeneity test (Cochran's Q) | | Pleiotropy test | MR PRESSO | | F |
|---|---|---|---|---|---|---|
| | P (MR Egger) | P(IVW) | P (MR Egger) | P(Global test) | Outlier | |

| | | | | | | |
|---|---|---|---|---|---|---|
| WMH volume | 0.24 | 0.18 | 0.15 | 0.23 | NA | 18.84 |
| FA | 0.05 | 0.07 | 0.82 | 0.11 | NA | 18.84 |
| MD | 0.06 | 0.08 | 0.89 | 0.08 | NA | 18.84 |
| lacunar stroke | 0.004 | 0.005 | 0.60 | 0.003 | NA | 20.00 |
| all location BMB | 0.65 | 0.70 | 0.69 | 0.80 | NA | 19.61 |
| lobar BMB | 0.89 | 0.92 | 0.67 | 0.74 | NA | 19.61 |
| mixed or deep BMB | 0.67 | 0.70 | 0.56 | 0.75 | NA | 19.61 |
| all location ICH or SVS | 0.18 | 0.16 | 0.25 | 0.21 | NA | 18.39 |
| lobar ICH or SVS | 0.05 | 0.06 | 0.44 | 0.09 | NA | 18.39 |
| non-lobar ICH or SVS | 0.40 | 0.28 | 0.09 | 0.29 | NA | 18.39 |
